# *In silico* Neuropeptidome of Female *Macrobrachium rosenbergii* Based on Transcriptome and Peptide Mining of Eyestalk, Central Nervous System and Ovary

**DOI:** 10.1371/journal.pone.0123848

**Published:** 2015-05-29

**Authors:** Saowaros Suwansa-ard, Tipsuda Thongbuakaew, Tianfang Wang, Min Zhao, Abigail Elizur, Peter J. Hanna, Prapee Sretarugsa, Scott F. Cummins, Prasert Sobhon

**Affiliations:** 1 Department of Anatomy, Faculty of Science, Mahidol University, Bangkok, Thailand; 2 Faculty of Science, Health, Education and Engineering, University of the Sunshine Coast, Maroochydore, Queensland, Australia; 3 Pro Vice-Chancellor’s Office, Faculty of Science, Engineering and Built Environment, Deakin University, Geelong, Victoria, Australia; University of Würzburg, GERMANY

## Abstract

*Macrobrachium rosenbergii* is the most economically important of the cultured freshwater crustacean species, yet there is currently a deficiency in genomic and transcriptomic information for research requirements. In this study, we present an *in silico* analysis of neuropeptide genes within the female *M*. *rosenbergii* eyestalk, central nervous system, and ovary. We could confidently predict 37 preproneuropeptide transcripts, including those that encode bursicons, crustacean cardioactive peptide, crustacean hyperglycemic hormones, eclosion hormone, pigment-dispersing hormones, diuretic hormones, neuropeptide F, neuroparsins, SIFamide, and sulfakinin. These transcripts are most prominent within the eyestalk and central nervous system. Transcript tissue distribution as determined by reverse transcription-polymerase chain reaction revealed the presence of selected neuropeptide genes of interest mainly in the nervous tissues while others were additionally present in the non-nervous tissues. Liquid chromatography-mass spectrometry analysis of eyestalk peptides confirmed the presence of the crustacean hyperglycemic hormone precursor. This data set provides a strong foundation for further studies into the functional roles of neuropeptides in *M*. *rosenbergii*, and will be especially helpful for developing methods to improve crustacean aquaculture.

## Introduction

Crustaceans are a group of invertebrates that encompass the second largest group of arthropods. The decapod crustaceans, including the freshwater prawns, lobsters, and crabs, are the most economically important crustacean aquaculture species, primarily due to their value as a food source. Advances in optimizing their culture can be made through a deeper understanding of regulation of growth and reproduction [1]. Therefore, many scientific studies concentrate on their growth and reproductive processes to ultimately obtain the tools to enable more efficient culture and supply of edible crustaceans. However, our knowledge and understanding of the molecular mechanisms involved in crustacean neuropeptides, which regulate many physiological activities, is still far from complete. This is partially due to decapod crustaceans having separate sexes and reproductive systems that may differ significantly [2, 3].

The eyestalk, central nervous system (CNS), and ovary are the primary tissues for studying the expression of genes and proteins involved in crustacean growth and reproduction. These tissues are known to be major sites for production and secretion of many hormones, for instance, allatostatin [4–6], corazonin [7], crustacean hyperglycemic hormones (CHHs) [8, 9], FMRF-amide [10, 11], and APGWamide [12]. Moreover, receptors involved in hormone pathways have been found to localise to these eyestalk, CNS and ovary, such as those receptors for allatostatin [13], neuropeptide F [9], pigment-dispersing hormone [9, 14], leptin [15, 16], and retinoid X receptor [17, 18]. Gene discovery has in recent times been helped by next-generation transcriptome sequencing, especially for the study of genes in selected tissues of non-model organisms that lack genomic information. This technique is now being applied widely as a shotgun method for creating accurate gene data. For example, transcriptomic results from muscle, ovary, and testis of *Macrobrachium rosenbergii* provided 123,534 transcripts, which were examined to provide a list of genes that may influence growth [19].

Other transcriptome studies have been forthcoming from a variety of crustaceans that have provided valuable information on the genetic makeup of specific tissues, and at different developmental stages. These include the Chinese mitten crab (*Eriocheir sinensis*) from which Illumina-derived testis transcriptomes were used to provide a list of genes required for development and spermatogenesis [20]. Also, the transcriptome analyses of the Pacific white shrimp (*Litopenaeus vannamei*), oriental river prawn (*Macrobrachium nipponense*), and amphipod (*Pahyale hawaiensis*) have enabled for deeper understanding of the molecular basis for embryonic development, gametogenesis, sex determination and differentiation [21–23]. Furthermore, the mining of neuropeptides by *in silico* analyses has been completed in a number of crustacean species [7, 24–26]. Several investigations have also confirmed the presence of neuropeptides in crustaceans by using mass spectrometry techniques [7, 27–31]. Cumulatively, these studies have greatly increased the number of decapod crustacean genes and proteins present within the public databases and provided some insight into the biology of non-model crustaceans where sequenced genomes have not been explored. However, use of this information for reliable control of growth and reproduction has not yet been implemented since functional analyses must be performed.

The giant freshwater prawn, *M*. *rosenbergii*, is found in tropical and subtropical zones, generally within freshwater ponds, rivers, lakes, canals, and estuarine areas. It is commercially important primarily due to its large size, low aggressive nature under culture conditions, and relative ease to culture. Insufficient genomic and transcriptomic resources are currently available for *M*. *rosenbergii*, restricting advances in aquaculture biotechnology, including what neuropeptides control their physiological activities, including growth, molting, feeding, and reproduction [32, 33]. In this study, we present the transcriptomes of eyestalk, CNS, and ovary from female *M*. *rosenbergii*. We present 37 prepropeptide transcripts that are common to crustaceans, with the majority being present within the eyestalk and CNS. Liquid chromatography-mass spectrometry analysis of eyestalk peptides has confirmed the presence of the crustacean hyperglycemic hormone precursor.

## Materials and Methods

### Animals and tissue collection

Live adult female *M*. *rosenbergii* were obtained from a local market (Phran Nok market, Bangkok, Thailand) and acclimatized for 24 h in a shrimp culture tank (Mahidol University) prior to tissue collection. The ovarian maturation stage for each individual was determined according to a previous study by Meeratana and Sobhon, 2007 [34]. Tissues, including eyestalks, CNS (pooled brain, thoracic ganglia, and abdominal ganglia), and ovaries, were collected from animals at various stages of ovarian maturation (ovarian stages I-IV; 20 prawns/stage; n = 80), and kept at -80°C until total RNA isolation and peptide extraction.

### RNA isolation, Illumina sequencing, and transcriptome assembly

Total RNA from *M*. *rosenbergii* eyestalks, CNS, and ovaries was isolated using TriPure isolation reagent (Roche, IN, USA) following the manufacturer's protocol. The quality and concentration of total RNA was checked by gel electrophoresis and spectrophotometry (NanoDrop 1000; Thermo Fisher Scientific, DE, USA). Twenty micrograms of total RNA of each tissue was dried before sending to BGI, Hong Kong, for library construction using their standard workflow for *de novo* RNA-seq transcriptomes (http://bgiamericas.com/). Briefly, for complementary DNA (cDNA) synthesis, RNA samples were subjected to oligo-dT selection for mRNA purification and fragmented into small fragments. Fragmented RNA samples were subsequently repaired before adapter ligation. The suitable fragments were selected and reversed-transcribed into double-stranded cDNAs. The cDNA libraries were normalized using a duplex-specific nuclease and constructed by PCR amplification using random hexamer primed cDNAs. Finally, the samples were sequenced using paired-end strategy and an Illumina HiSeq 2000 instrument (Illumina Inc.). Raw reads from sequencing were then filtered in order to remove those reads that contain adaptor, unknown nucleotides (>5%), or low quality reads (>20% of low quality bases). *De novo* assemblies were performed individually and combined by SOAPdenovo software [35] using trimmed reads. The assembler was run with the parameters set as follows: seqType, fq; minimum kmer coverage = 4; minimum contig length of 100 bp; group pair distance = 250. Non-redundant assembled sequences were defined as unigenes of which expression levels were calculated using the FPKM method (Fragments Per kb per Million fragments) [36]. The formula is FPKM = (1000000*C)/(N*L*1000). Assigned FPKM(A) to be the expression of gene A, C to be number of fragments that uniquely aligned to gene A, N to be the total number of fragments that uniquely aligned to all genes, and L to be the number of bases on gene A.

### Transcriptome analysis and *in silico* mining of neuropeptides and their receptors

For transcriptome analysis, all unigenes assembled from combined three-transcriptome dataset (eyestalk, CNS, and ovary) were annotated with the databases of non-redundant protein sequence (Nr), nucleotide sequence (Nt), Swiss-Prot, Kyoto Encyclopedia of Genes and Genomes (KEGG), Clusters of Orthologous Groups (COG), and Gene Ontology (GO), using BLAST and BLAST2GO softwares. Furthermore, individual transcriptome analysis was carried out to examine the expression pattern of unigenes among three transcriptomes using gene identification (ID) mapping. All unigenes from ID mapping were then annotated against Pfam databases (version 27.0) using HMMER software [threshold of e-value = 0.01) [37]. Searches with arthropod neuropeptides and receptors were also conducted by performing tBLASTn searches of all unigenes assembled from combined dataset against other known peptides and receptors, which were reported by previous-omics studies [5, 25, 38–40]. BLAST searches were performed using the CLC Main Workbench Version 6.0 [41]. All hits were analyzed manually with their orthologous peptides from various species based on sequence alignments and predicted cleavage products. Then, protein characterization was carried out based on previously known orthologous peptides.

### Protein prediction and phylogeny

Analysis of sequence identity/similarity between different proteins was performed by protein alignment using ClustalW2 (http://www.ebi.ac.uk/Tools/msa/clustalw2/) [42]. The percent identity was calculated as the number of identical amino acid residues, as indicated by "*" symbol in the Clustal output, divided by the total number of amino acid residues of the longest sequence (x100). The percent of similarity was calculated from the total number of identical and similar amino acid residues (as denoted by ":" and "." symbols) divided by the total number of amino acids of the longest sequence (x100). Prediction of receptor transmembrane domains was performed using the PredictProtein online tool (https://www.predictprotein.org/) [43]. All hits (lowest E-value) were run through the SignalP 4.1 for signal peptide prediction with the Neural Networks algorithm (Center for Biological Sequence Analysis, Technical University of Denmark, Lyngby, Denmark; http://www.cbs.dtu.dk/services/SignalP/) [44]. Proteolytic cleavage sites as well as post-translational modifications were predicted based on homology to other known peptides using NeuroPred application (http://neuroproteomics.scs.illinois.edu/cgi-bin/neuropred.py) [45].

Full-length peptide precursors were analyzed for their evolutionary relationship with other known orthologous peptide precursors, which was represented by a phylogenetic tree using the MEGA 5 program with parameters set as follows: statistical method, maximum likelihood; amino acid substitution model, Jones-Taylor-Thornton (JTT) with frequencies [46]. Tree branches are indicated by the abbreviated species name followed by the GenBank accession number. For species abbreviations and all Genbank accession numbers of peptides used in sequence alignments, see Tables A and B in S1 Table.

### Tissue distribution of selected genes of interest by reverse transcription-polymerase chain reaction (RT-PCR)

Eyestalk, brain, thoracic ganglion, abdominal ganglion, ovary (stages I-IV), hepatopancreas, stomach, gill, heart, and muscle tissues were collected from female prawns (n = 10). In addition, testes were obtained from male prawns and used to check whether there were any sex-specific gene transcripts. Total RNA was extracted using TriPure isolation reagent (Roche, IN, USA) and following the manufacturer's protocol. Two micrograms of total RNA per tissue was used for cDNA synthesis using a Tetro cDNA synthesis kit (Bioline, UK). Gene-specific primers were designed from the transcriptome-derived nucleotide sequences using Primer-BLAST software [47] and PCR was carried out following a routine protocol optimized for individual genes (Table 1). PCR products were analyzed by agarose gel electrophoresis and amplicons purified (QIAquick gel extraction kit, Qiagen, Germany) for sequencing.

**Table 1:** Gene-specific primers and expected amplicon sizes.

Genes	Forward primer (5'→3')	Reverse primer (5'→3')	Amplicon size (bp)
Bursicon-α	TGTCAGCAACAATGACGAGC	ACAGGATCTTTCCGTCTGCC	224
Bursicon-β	CGGGAAGATGTGGTCCTTGT	AGGGCTGTACCTTCGACAGA	221
CLDH	ACAACTCGGCTCTGGTGTTC	CGTCGTCGGAACTTCTCCTC	309
CCAP	TCATCGTCGTAGCGATGCAG	GAGCGCATGACGTCCATCTT	241
Eclosion hormone	TCAGCGTTGTTTGCTTGGTC	TTTCGATTCAGGTCATGCGGA	394
Neuropeptide-F-I	CCAAGTGTGGGCGGCTATTT	TCACCAGGAGGAACGGCATA	201
Neuropeptide-F-III	ATACAAGGCGCAACAACACC	GTCGTCGTGTGAGGATGACC	332
Neuroparsin-1	GCCCTAAACATTCCCCCGAC	GCAATATGTACCTTCGCCGC	356
Neuroparsin-2	TGAAGTCGTTTGCTGCTTGC	AGCAGTGATCTTTCCGGGTG	332
SIFamide	GTCAGAGTCAACCACACGGA	CCGGATTCGTATGCAGGGTC	227
Sulfakinin	TCTGTGACACGACCCTCCTC	TACTCGTCGAACTGTCGCTT	260

Annealing temperature for RT-PCR was 60°C for all gene amplifications.

Abbreviation: CLDH, Calcitonin-like diuretic hormone; CCAP, Crustacean cardioactive peptide.

### Peptide isolation and liquid chromatography-mass spectrometry (LC-MS/MS)

Total proteins were extracted from the eyestalks of female *M*. *rosenbergii* at all stages of ovarian maturation. Briefly, tissues were collected from animals (n = 80) and kept at -80°C until use. Total proteins were extracted using lysis buffer (150 mM NaCl, 1.0% Triton X-100, 50 mM Tris, 1 mM EDTA, 1 mM PMSF, pH 8.0). To purify peptides, extracts were filtered through an Ultrafree-MC, HV 0.45 μm pore-size sterile filter (Millipore Corporation, Billerica, MA, USA). Then, extracts were centrifuged through a Centricon-10 molecular weight cut-off concentrator (10 kDa; Millipore Corporation, Billerica, MA, USA) at 5000 *×g* for 30 min. The flow-through was collected and lyophilized before further analysis. For protein separation, protein samples were dissolved in 0.1% trifluoroacetic acid (TFA) in milliQ water. Samples were then separated by reverse phase high-performance liquid chromatography (RP-HPLC) using a Zorbax 50x0.5 mm C18 column (Agilent). The mobile phases for reverse phase separation were 0.1% TFA in MilliQ water (solution A) and 0.1% TFA in acetonitrile (solution B). The gradient profile was set as follows: increasing linear gradient from 5% to 95% of solution B; flow rate = 2 ml/min; run time = 60 min. HPLC fractions were collected and lyophilized for LC-MS/MS analysis.

Samples were resuspended in 0.1% formic acid, then LC-MS/MS was performed on a Shimadzu Prominance Nano HPLC (Japan) coupled to a Triple Tof 5600 mass spectrometer (ABSCIEX, Canada) equipped with a nano electrospray ion source. A 6 μL sample of each extract was injected onto a 50 mm x 300 μm C18 trap column (Agilent Technologies, Australia) at 30 μL/min. The samples were de-salted on the trap column for 5 min using 0.1% formic acid (aq) at 30 μL/min. The trap column was then placed in-line with the analytical nano HPLC column, a 150 mm x 75 μm 300SBC18, 3.5 μm (Agilent Technologies, Australia) for mass spectrometry analysis. Linear gradients of 1–40% solvent B over 35 min at 300 nL/min flow rate, followed by a steeper gradient from 40% to 80% solvent B in 5 min were used for peptide elution. Solvent B was held at 80% for 5 min for washing the column and returned to 1% solvent B for equilibration prior to the next sample injection. Solvent A consisted of 0.1% formic acid (aq) and solvent B contained 90/10 acetonitrile/0.1% formic acid (aq). The ion spray voltage was set to 2400 V, declustering potential (DP) 100 V, curtain gas flow 25, nebuliser gas 1 (GS1) 12 and interface heater at 150°C. The mass spectrometer acquired 500 ms full scan TOF-MS data followed by 20 by 50 ms full scan product ion data in an Information Dependent Acquisition (IDA) mode. Full scan TOFMS data was acquired over the mass range 350–1800 and for product ion ms/ms 100–1800. Ions observed in the TOF-MS scan exceeding a threshold of 100 counts and a charge state of +2 to +5 were set to trigger the acquisition of product ion, ms/ms spectra of the resultant 20 most intense ions. The data was acquired and processed using Analyst TF 1.5.1 software (ABSCIEX, Canada).

Peptides were identified by database searching using PEAKS v7.0 (BSI, Canada) against the protein database built from the eyestalk transcriptome. Search parameters were as follows: no enzyme was used; and variable modifications included methionine oxidation, conversion of glutamine to pyroglutamic acid, deamidation of asparagine and amidation. Precursor mass error tolerance was set to 20 ppm and a fragment ion mass error tolerance was set to 0.05 Da. The maximum expectation value for accepting individual peptide ion scores [-10*Log(*p*)] was set to ≤0.05, where *p* is the probability that the observed match is a random event. Proteins and their supporting peptides were obtained and analyzed.

## Results

### *M*. *rosenbergii* transcriptome assembly and annotation

Assembled sequence data was prepared from female *M*. *rosenbergii* RNA obtained from eyestalks, CNS and ovaries from prawns at different ovarian stages. Clean reads passed through basic quality standards were clustered and assembled *de novo*. Illumina high-throughput generation sequencing from eyestalk generated a total of 52,340,948 clean reads representing a total of 4,710,685,320 (4.71 Gb) nucleotides. For CNS sequencing, a total of 54,259,826 clean reads were obtained, providing a total of 4,883,384,340 (4.88 Gb). For ovary sequencing, a total of 51,563,078 clean reads were obtained, providing a total of 4,640,677,020 (4.64 Gb) nucleotides. Assembly of each tissue transcriptome created 65,342, 60,482, and 60,055 unigenes from eyestalk, CNS and ovary, respectively, whereas assembly of a combined three-tissue transcriptome generated 78,110 unigenes. An overall summary of sequencing outcomes is provided in S2 Table, which also includes the number of total raw reads, percentages of Q20 and GC, as well as length of unigenes in each transcriptome dataset. All sequence data can be found in the NCBI database (accession code SRP049917).

**Fig 1:**
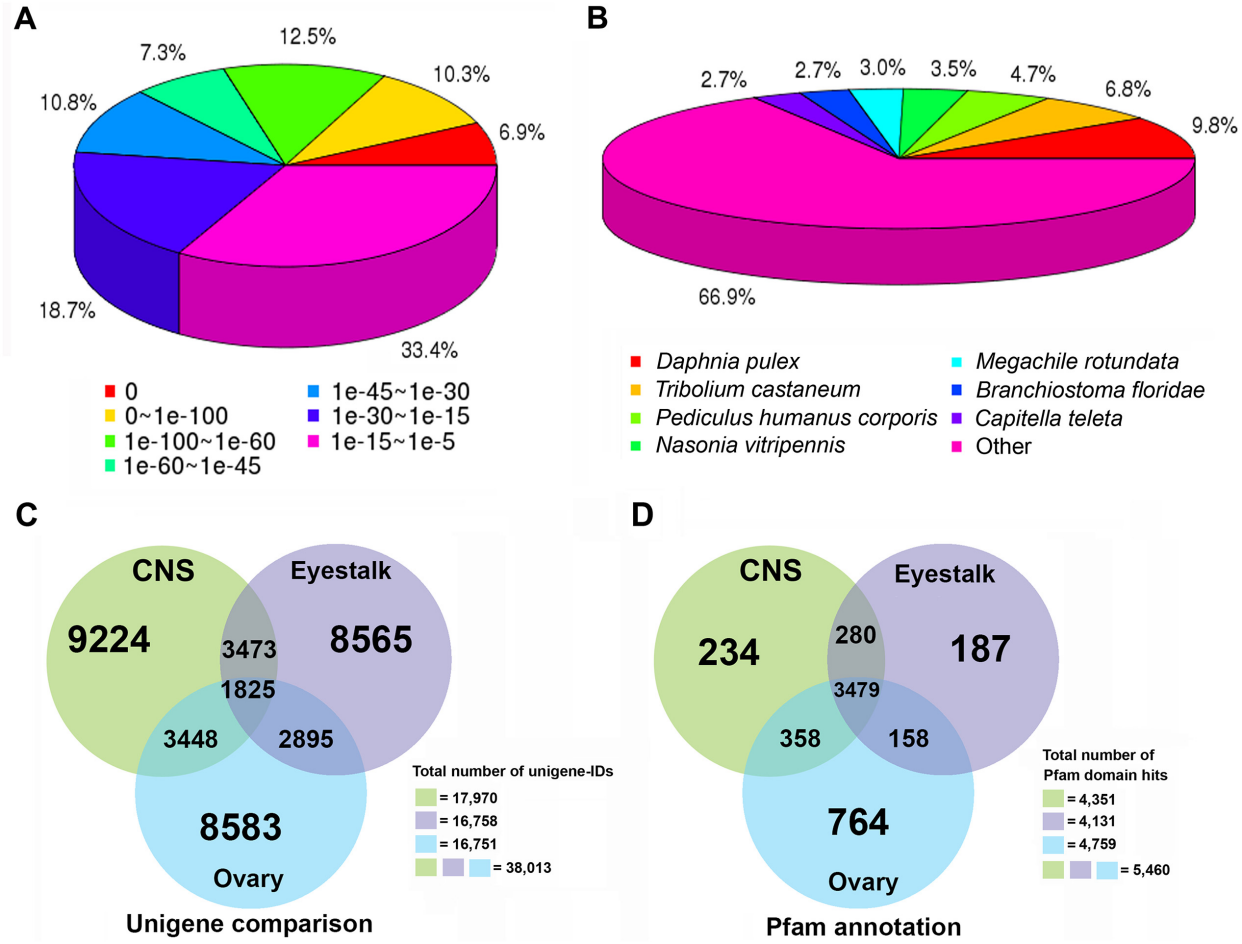
Summary of BLAST searches in combined three-transcriptome dataset and unigene analyses in individual datasets (eyestalk, CNS, and ovary) of *Macrobrachium rosenbergii*. (A) E-value distribution of BLASTx hits from all unigenes obtained within combined eyestalk, CNS, and ovary dataset. (B) Species distribution of BLASTx hits. (C) Comparison of unigenes present in individual datasets. (D) Pfam annotation of unigenes present in individual datasets.

For gene annotation, all unigenes assembled from the combined three-transcriptome dataset were subjected to searches with BLASTx against the NCBI Nr and Swiss-Prot databases. Of the 78,110 unigenes, 28,336 unigenes (36.3%) returned hits with databases at a cut-off value 1x 10e^-5^. The E-value distribution of these is shown in Fig 1A; species most represented in the BLASTx searches included *Daphnia pulex* (9.8%), *Tribolium castaneum* (6.8%), *Pediculus humanus corporis* (4.7%), while "other species" was the largest group (66.9%) (Fig 1B). From gene ID mapping, we could identify 38,013 unigene-IDs from which 16,758, 17,970, and 16,751 unigene-IDs were present in the eyestalk, CNS, and ovary, respectively. Unigene comparison between the three transcriptomes showed 1,825 common unigene-IDs while the numbers of tissue-specific unigenes showed 8,565 for eyestalk, 9,224 for CNS, and 8,583 for ovary (Fig 1C). In total, there were 12,299 eyestalk, 13,730 CNS, and 12,840 ovary unigene-IDs (data not shown) annotated with 5,460 unique Pfam domains. The total number of Pfam domain hits in the eyestalk, CNS, and ovary transcriptomes were 4,131, 4,351, and 4,759 Pfam domains, respectively. Over 64% of the Pfam domains (3,479 unigene-IDs, Fig 1D) are common in the three tissues.

### Clusters of Orthologous Groups (COG) classification and Gene Ontology (GO) assignments

Functional annotation using the COG database classified 9,853 unigenes into 25 categories as shown in S1A Fig. The largest represented biological process falls within the "general function" prediction category (49.72%), followed by translation, ribosomal structure and biogenesis category (24.08%), and replication, recombination and repair category (22.06%). There were 1,632 unigenes (16.56%) that could not be placed into any biological process category. A total of 9,357 unigenes were assigned for GO terms based on BLAST matches with sequences of proteins with known functions in terms of biological process, molecular function, and cellular component. A distribution of the unigenes in different GO categories is shown in S1B Fig. Unigene sequences were assigned to cellular components (32,166 sequences), molecular functions (16,805 sequences), and biological processes (59,730 sequences). The cellular process, single organism process, and metabolic process were most highly represented for biological processes. For cellular component, genes involved in the cell and cell part categories were the most represented GO assignments, followed by organelle category. In addition, the most well represented assignments within the molecular function category were the binding (50%) and catalytic activities (46%). Within the biological processes category, about 9% of unigenes were involved in either a reproductive process or reproduction.

### Characterization of predicted neuropeptide and neuropeptide receptor transcripts of interest in *M*. *rosenbergii*

Sequence and annotation information for crustacean neuropeptides and their receptors (if known) are provided in Tables 2–4, S1 and S2 Files. Further transcript characterization was performed for selected neuropeptides that were deemed to play an important role in crustacean physiological activities, including molting, feeding, metabolism, growth, and reproduction.

**Table 2:** Summary of transcripts encoding neuropeptides in *M*. *rosenbergii* eyestalk, CNS, and ovary transcriptomes.

Neuropeptides	Precursor size/Signal peptide (amino acids)	Es (FPKM value)	CNS (FPKM value)	Ov (FPKM value)	E-value	BLAST hit neuropeptide and species	Accession numbers
AST-A	614/27	30.51	135.11	0	0.00E+00	AST-A [*M*. *rosenbergii*]	AAY82901
AST-B	375/25	67.99	132.29	0	6.00E-92	AST-B-1 [*P*. *japonica*]	AFV91538
Bur-α	147/26	0	98	0	2.00E-76	Bur-α subunit [*H*. *gammarus*]	ADI86242
Bur-β	136/21	0	141.91	0	1.00E-70	Bur-β subunit [*H*. *gammarus*]	ADI86243
CLDH	134/20	11.83	45.96	0	8.00E-51	CLDH [*H*. *americanus*]	ACX46386
DH45	250/20	1.86	5.25	0	0.018	DH44 [*P*. *americana*]	P41538
Crz	76/24	8.88	0	0	4.00E-07	Corazonin [*D*. *pulex*]	ACJ05606
CCAP	142/28	88.66	1025.2	0	9.00E-44	CCAP [*P*. *clarkii*]	BAF34909
CFSH-1	209/29	15.5	0	0	1.00E-07	CFSH [*C*. *maenas*]	AEI72264
CFSH-2	233/32	48.33	0	0	1.00E-30	CFSH [*C*. *maenas*]	AEI72264
CHH-1	135/26	231.7	3.69	0	0.00E+00	CHH [*M*. *rosenbergii*]	AAL40915
CHH-2	134/26	3.53	1.93	0	0.00E+00	CHH-L [*M*. *rosenbergii*]	AAL40916
CHH-3	152/18	26.8	0	0	2.00E-37	CHH [*P*. *japonica*]	AFG16933
EH	82/26	170.1	31.33	0	4.05E-17	EH-2 [*N*. *lugens*]	BAO00951
FLRFamide-I	364/18	29.49	40.16	0	2.00E-26	FLRFamide-A [*P*. *clarkii*]	BAE06262
FLRFamide-II	180/18	0	25.742	0	9.00E-06	FLRFamide-A [*P*. *clarkii*]	BAE06262
MIH-A	119/41	47.86	17.13	0	1.00E-82	MIH-A [*M*. *rosenbergii*]	AAL37948
MIH-B	112/34	192.6	0	0	1.00E-76	MIH-B [*M*. *rosenbergii*]	AAL37949
Myosuppressin	101/31	204.6	191.96	0	1.00E-37	Myosuppressin [*P*. *clarkii*]	BAG68789
NP-1	100/26	6.37	10.45	89.9	4.47E-31	Neuroparsin [*M*. *ensis*]	AHX39208
NP-2	99/24	153.6	460.07	6.09	3.79E-09	Neuroparsin [*M*. *ensis*]	AHX39208
NPF-I	90/29	29.15	56.31	0	3.00E-30	NPF-I [*L*. *vannamei*]	AEC12204
NPF-II	127/29	5.12	20.06	0	4.00E-44	NPF-II [*L*. *vannamei*]	AEC12205
NPF-III	104/23	75.13	44.9	0	4.00E-11	Neuropeptide Y [*L*. *stagnalis*]	CAB63265
sNPF	170/25	53.33	41.91	0	2.00E-05	sNPF [*A*. *aegypti*]	ABE72968
Orcokinin	84/21	570.7	1663.6	0	3.00E-27	Orcokinin [*P*. *clarkii*]	Q9NL83
OT/VP-like	146/19	33.54	26.8	0	5.00E-26	Vasotocin [*P*. *shermani*]	ABP88922
PDH-α-1	77/21	22.18	0	0	1.00E-18	PDH-related peptide [*Penaeus* sp.]	JC4756
PDH-α-2	79/23	40.24	0	0	2.00E-18	PDH type-2 [*L*. *vannamei*]	P91964
PDH-α-3	80/24	89.91	0	0	8.00E-18	PDH type-2 [*L*. *vannamei*]	P91964
PDH-α-4	79/23	28.74	0	0	2.00E-18	PDH type-2 [*L*. *vannamei*]	P91964
PDH-α-5	77/21	161.5	0	0	2.00E-18	PDH-related peptide [*Penaeus* sp.]	JC4756
PDH-β	80/22	667.1	134.36	0	5.00E-28	PDH-related peptide [*Penaeus* sp.]	JC4756
RPCH	97/21	192.5	96.48	0	2.00E-49	RPCH [*M*. *rosenbergii*]	ABV46765
SIFamide	76/27	346.1	609.29	0	1.88E-28	SIFamide [*M*. *rosenbergii*]	CDG15340
Sulfakinin	126/32	16.1	5.87	0	3.00E-29	Sulfakinin [*H*. *americanus*]	ABQ95346
TRP	210/25	5.45	22.6	0	4.00E-84	Tachykinin-A [*P*. *interruptus*]	BAD06362

Abbreviations: AST, Allatostatin; Bur, Bursicon; CLDH, Calcitonin-like diuretic hormone; DH45, Diuretic hormone-45; Crz, Corazonin; CCAP, Crustacean cardioactive peptide; CFSH, Crustacean female sex hormone; CHH, Crustacean hyperglycemic hormone; EH, Eclosion hormone; MIH, Molt inhibiting hormone; NP, Neuroparsin; NPF, Neuropeptide F; sNPF, Short Neuropeptide F; OT, Oxytocin; VP, Vasopressin; PDH, Pigment dispersing hormone; RPCH, Red pigment-concentrating hormone; TRP, Tachykinin-related peptide; Es, eyestalk; CNS, central nervous system; Ov, ovary; FPKM, Fragments Per kb per Million fragments.

The total length of precursor, number of predicted signal peptide residues, transcription sites with FPKM value, lowest E-value, BLASTx hits, and Genbank accession number of BLAST hit peptides are indicated.

**Table 3:** Predicted mature peptides from the eyestalk, CNS, and ovary transcriptomes of *M*. *rosenbergii*.

Neuropeptides	Predicted mature peptides	Remarks
AST-A (Partial)	HNDYVFGLa	Mro-AST-A-1
	SPGYSFGLa	Mro-AST-A-2
	DRTYSFGLa	Mro-AST-A-3
	EGLYAFGLa	Mro-AST-A-4
	SGTYNFGLa	Mro-AST-A-5
	GQYAFGLa	Mro-AST-A-6
	SKAFSFGLa	Mro-AST-A-7
	DRSYSFGLa	Mro-AST-A-8
	SQQYAFGLa	Mro-AST-A-9
	PRHYAFGLa	Mro-AST-A-10
	PSSYAFGLa	Mro-AST-A-11
	PKNYAFGLa	Mro-AST-A-12
	DSDIQTRPGQYAFGLa	Mro-AST-A-13
	PQHYAFGLa	Mro-AST-A-14
	PQQYAFGLa	Mro-AST-A-15
	PQHYAFGLa	Mro-AST-A-16
	PQHYAFGLa	Mro-AST-A-17
	PQHYAFGLa	Mro-AST-A-18
	PQHYAFGLa	Mro-AST-A-19
	AQQYAFGLa	Mro-AST-A-20
	ASSYSFGLa	Mro-AST-A-21
	AGQYAFGLa	Mro-AST-A-22
	GGPYAFGLa	Mro-AST-A-23
	SPYSFGLa	Mro-AST-A-24
	PDAYSFGLa	Mro-AST-A-25
	SRTYQFGLa	Mro-AST-A-26
	AGPYTFGLa	Mro-AST-A-27
	ENQYAFGLa	Mro-AST-A-28
	AGHYSFGLa	Mro-AST-A-29
	SSPYAFGLa	Mro-AST-A-30
	SRPYAFGLa	Mro-AST-A-31
AST-B	ADWSSMRGTWa	Mro-AST-B-1
	AGWNKFQGSWa	Mro-AST-B-2
	ANWNKFQGSWa	Mro-AST-B-3 (3 copies)
	GNWKNFQGSWa	Mro-AST-B-4 (2 copies)
	NSWSSLQGSWa	Mro-AST-B-5
	AGWSSLQGSWa	Mro-AST-B-6
	AWKNLHGAWa	Mro-AST-B-7
	STNWSSLRGTWa	Mro-AST-B-8
	SADWSSLRGAWa	Mro-AST-B-9
	NTDWSQFRGSWa	Mro-AST-B-10
Bur-α	DECSLTPVIHILSYPGCNSKPIPSFACQGRCTSYVQVSGSKIWQTERSCMCCQESGEREATVTLNCPKARVGDPKRRKVLTRAPVDCMCRPCTDIEEGTVLAQEIANFIADDPMAHVPFLK	Mro-Bur-α
Bur-β	KGYRAECETLPSTVHVSKEEYDEAGRLLRTCEEDLAVNKCEGACLSKVQPSVNTPSGFLKDCRCCRETHLRSREVILTHCYDVDGNRLTGEKGQLSLKLSEPADCQCAKCGDSTR	Mro-Bur-β
CLDH	GLDLGLGRGFSGSQAAKHLMGLAAANFAGGPa	Mro-CLDH (DH31)
	AVVEIDDPDY[SO3H]VLELLTRLGHSIIRANELENA	Mro-CLDH precursor-related peptides
	SSDDAANTTHDLHHLEDNY[SO3H]AQEPAAVDSAAAASR	Mro-CLDH precursor-related peptides
DH45	SSGLSLSIDASMKVLREALYLEMARKKQRQQMQRARHNQELLTSIa	Mro-DH45
	LPLEGVRSAPHQQQQPTLLEDLSPNNLPPQDFTQDDY[SO3H]QQLLTRQPDSAAAALNSYDASETLPYMYRLQEGLAGAGLPATGLDGDVTRLENPDWMAVDPRSYLLAQFLEHPAEEATGSESNSMIPIRKI	Mro-DH45 precursor-related peptide
Crz (partial)	pQTFQYSRGWTNa	Mro-Crz
	SDPTIGQRKGVDNMIQTLPVSRLLAEGAPHQHGGSAR+	Mro-Crz precursor-related peptide
CCAP	PFCNAFTGCa	Mro-CCAP
	SPVA	Mro-CCAP precursor-related peptides
	DIGGLLEGKD	Mro-CCAP precursor-related peptides
	SDASVEALASGTELDDLAKHVLAEAKLWEQLQNKMDVMRSLAARMDEQRPLY	Mro-CCAP precursor-related peptides
	SPAAAAAAPEPRQQLAASSQQQQTKTQ	Mro-CCAP precursor-related peptides
CFSH-1	GQVSMIPANQVKQGWEEDYTSVPDVLIQFSQKQAEETVCNDLSVQLFRVDLSEHYLEPVWVKEIVHLGMCPSKLQTRSFGKDVWPSTIVEAKCLCNNQPCSNLGGDFRCQAVRKPIRTWVRHVEKFMPVQEMVTVGCVCVQRTSPEGKYARPAIEA	Mro-CFSH-1
	TRAPPTFV	Mro-CFSH-1 precursor-related peptides
	AKTCANQNQSRC	Mro-CFSH-1 precursor-related peptides
CFSH-2	KQEDLSTDELQYFSEEQVDEASRVEYKVVPDPVIYTSQIIHKGVNCSSIKTDLHKNHITPELQLHPEWIHTSQLIGTCPTHYVTRELPPMYSPSVVVEAVCTCTGSKCSRDGHQCLPVSRHIPVWVRQGPNFHVLDVEELTVACACV	Mro-CFSH-2
	TLSVPSYQQPFLGSGWEETIPKHMKWSDEAIRIM	Mro-CFSH-2 precursor-related peptides
	PSVGGNFIFASAVHSK	Mro-CFSH-2 precursor-related peptides
CHH-1	AILDQSCKGIFDRELFKKLDRVCDDCYNLYRKPYVAIDCREGCYQNLVFRQCIQDLQLMDQLDEYANAVQIVa	Mro-CHH-1
	WSVDGLARIEKLLSTSSSASAASPTRGQALNLK	Mro-CHH-1 precursor-related peptide
CHH-2	AILDQSCKGIFDRELFKKLDRVCDDCYNLYRKPYVAIDCRKDCFGTKTFGHCVEDLLLDQTHYKEIRDHIALF	Mro-CHH-2
	WSVDGLARIEKLLSTSSSASAASPTRGQALNLK	Mro-CHH-2 precursor-related peptide
CHH-3	AAIDRSCKGIYDRGIFMMLDRVCEDCYNLYRKPYVGVDCRKRCYRNATFRQCLNDLLLKDNFDSYADLVRTVa	Mro-CHH-3
	LGTTSTQAVASGATDSLPGIETLFSSSFSSSLASTSPNAQPPPPPPSLLESLRGHGAD	Mro-CHH-3 precursor-related peptide
EH	ASITSMCIRNCGQCKEMYGDYFHGQACAESCIMTQGVSIPDCNNPATFNRFLKRFI	Mro-EH
FLRFamide-I	GYGDRNFLRFa	Mro-FLRFamide-I-a
	DGGRNFLRFa	Mro-FLRFamide-I-b
	DRNFLRFa	Mro-FLRFamide-I-c (3 copies)
	ADKNFLRFa	Mro-FLRFamide-I-d
	NYDKNFLRFa	Mro-FLRFamide-I-e
	PYSRDFLRFa	Mro-FLRFamide-I-f
FLRFamide-II	SSGRNFLRFa	Mro-FLRFamide-II-a
	NRNFLRFa	Mro-FLRFamide-II-b
	SRNFLRFa	Mro-FLRFamide-II-c
	GNRNFLRFa	Mro-FLRFamide-II-d
	QYNKNFLRFa	Mro-FLRFamide-II-e
	GNVDRSFIRFa	Mro-FLRFamide-II-f
MIH-A	RYLDDECPGVMGNRDLYEKVVRVCDDCSNIFRMNDMGTRCRKDCFYNVDFLWCVYATERHGDVDQLNRWMSILRAa	Mro-MIH-A
MIH-B	RFLDDECRGVMGNRDLYEYIVRICDDCENLFRKSNVGSRCKKNCFYNEDFMWCVRATERTDELEHLNRAMSIIRVa	Mro-MIH-B
Myosuppressin	QDLDHVFLRFa	Mro-Myosuppressin
	MPPPICTDQKLPLSPYAQKLCAALNNIAEFSRAMEEY[SO3H]LDAKAIKNSMPINEPEV	Mro-Myosuppressin precursor-related peptide
NP-1	APSCSTRRQQVNVETCKYGTYVDWCRNTVCAKGPGQSCGGDWWEYGKCGEGTYCACGTCSGCSLNLECWSGTFC	Mro-NP-1
NP-2	APRCTQHDRPPPEKCTYGTVLDWCRNEVCAKGPGETCGGHFWEQGKCGEGTFCSCGTCTGCSVITRRCFRSALVC	Mro-NP-2
NPF-I	KPDPTQLAAMADALKYLQELDKYYSQVSRPRFa	Mro-NPF-I
	SEYAVPPGDVLMEASERLMETLARRR	Mro-NPF-I precursor-related peptide
NPF-II	KPDPTQLAAMADALKYLQELDKYYSQVSRPSPRSAPGPASQIQALEKTLKFLQLQELGKLYSLRARPRFa	Mro-NPF-II
	SEYAVPPGDVLMEASERLMETLARRR	Mro-NPF-II precursor-related peptide
NPF-III	ARTGNAAETLQAMREADLAGILGSAEVPYPSRPNIFKSPVELRQYLDALNAYYAIAGRPRFa	Mro-NPF-III
	GGAMPQRSSSHDDLLDY	Mro-NPF-III precursor-related peptide
sNPF	GGGSIRSWQQVSQRSEPSLRLRYa	Mro-sNPF-1
	DRTPALRLRFa	Mro-sNPF-2
	APALRLRFa	Mro-sNPF-3
	GGGGGPPSMRLRFa	Mro-sNPF-4
	VPTPPDYDAALSDMYDLLSHGVE	Mro-sNPF precursor-related peptides
	TVDEAEPLLDHDLVRK	Mro-sNPF precursor-related peptides
	TSSDY[SO3H]LQDDAYDAADFIRQD	Mro-sNPF precursor-related peptides
	DVSYGQDEEASTGSHEQ	Mro-sNPF precursor-related peptides
Orcokinin (partial)	NFDEIDRSGFGFA	Mro-[Ala-13]Orcokinin
	FDSFTTGFGHS	Mro-Orcomyotropin
	NFDEIDRS+	possibly partial of Mro-Orcokinin
	GPVKTTANRAAATQQDPGYTDNAPV	Mro-Orcokinin precursor-related peptide
OT/ VP-like	CFITNCPPGa	Mro-OT/ VP-like peptide
	SMPSSHIGHTRTCTSCGPGLQGRCLGPEICCGETIGCFLGTRESQICRTENLIPVTCNNSDLKPCGVSRSGHCSAVGLCCTEAKCEFDINCISEGSQIa	Mro-Neurophysin-like
PDH-α-1	NSGMINSILGIPRVMAEAa	Mro-PDH-α-1
	QEDFPTTERKVVADLAAQILRVAHGPWSAAEAH	Mro-PDH-α-1 precursor-related peptide
PDH-α-2	NSGMINSILGIPRVMAEAa	Mro-PDH-α-2
	QEDLMTTERQVVAELAAQILRVAHGPWSAAEAH	Mro-PDH-α-2 precursor-related peptide
PDH-α-3	NSGMINSLLGIPMVMAEAa	Mro-PDH-α-3
	QEDLAATERQIVAELAAQILRVAHGPWSAAEAH	Mro-PDH-α-3 precursor-related peptide
PDH-α-4	NSGMINSLLGIPMVMAEAa	Mro-PDH-α-4
	QEDLMTTERQVVAELAAQILRVAHGPWSAAEAH	Mro-PDH-α-4 precursor-related peptide
PDH-α-5	NSGMINSLLGIPMVMAEAa	Mro-PDH-α-5
	QEDFPTTERKVVADLAAQILRVAHGPWSAAEAH	Mro-PDH-α-5 precursor-related peptide
PDH-β	NSELINSLLGLPKVMTDAa	Mro-PDH-β
	pQEELKYPERQVVAELAAQILRIA	Mro-PDH-β precursor-related peptides
	GPWGTVAAGTH	Mro-PDH-β precursor-related peptides
RPCH	pQLNFSPGWa	Mro-RPCH
	AAAAGVGTGSEAQLHSASGLALPGSSVTRGDNCASMQISTVMHIYRLI	Mro-RPCH precursor-related peptides
	pEASRLVQCQDEEY[SO3H]LA	Mro-RPCH precursor-related peptides
SIFamide	GYRKPPFNGSIFa	Mro-SIFamide
	SGGDPAYESGKTLASICQVAVEACSAWFPGPE	Mro-SIFamide precursor-related peptide
Sulfakinin	pQFDEY[SO3H]GHMRFa	Mro-Sulfakinin-I
	GGGAEY[SO3H]DDY[SO3H]GHLRFa	Mro-Sulfakinin-II
	APSKPSLALA	Mro-Sulfakinin precursor-related peptides
	LAPAIRQKLEESHMSPALMEEIVTDFEDPELMDFYDAE	Mro-Sulfakinin precursor-related peptides
	SLGRD	Mro-Sulfakinin precursor-related peptides
	TA	Mro-Sulfakinin precursor-related peptides
	HF	Mro-Sulfakinin precursor-related peptides
TRP	APSGFLGMRa	Mro-TRP (6 copies)

Abbreviations: AST, Allatostatin; Bur, Bursicon; CLDH, Calcitonin-like diuretic hormone; DH45, Diuretic hormone-45; Crz, Corazonin; CCAP, Crustacean cardioactive peptide; CFSH, Crustacean female sex hormone; CHH, Crustacean hyperglycemic hormone; EH, Eclosion hormone; MIH, Molt inhibiting hormone; NP, Neuroparsin; NPF, Neuropeptide F; sNPF, Short Neuropeptide F; OT, Oxytocin; VP, Vasopressin; PDH, Pigment dispersing hormone; RPCH, Red pigment-concentrating hormone; TRP, Tachykinin-related peptide; a, amidated C-terminus; Y[SO3H], sulfated tyrosine; pQ/pE, pyroglutamic acid; +, partial sequence with additional unknown amino acids.

Mro-AST-A mature peptides were predicted following previous work by Yin et al. (2006) [125].

**Table 4:** Summary of transcripts encoding neuropeptide receptors in *M*. *rosenbergii* eyestalk, CNS, and ovary transcriptomes.

Neuropeptide receptors	Size (amino acids)	Es (FPKM value)	CNS (FPKM value)	Ov (FPKM value)	E-value	BLAST hit receptor and species	Accession numbers
ACP receptor	244	6.07	3.77	4.6	2.00E-67	ACP receptor [*T*. *castaneum*]	NP001280549
Neuropeptide/AST-C receptor	67	3.51	0	0	1E-07/4E-07	Neuropeptide receptor A1 [*D*. *plexippus*]/AST-C receptor [*R*. *prolixus*]	EHJ63490/AHE41430
AST-A receptor	453	14.37	23.98	0	3.00E-10	AST receptor [*P*. *americana*]	AAK52473
DH receptor	115	4.44	0	0	4.00E-24	DH receptor [*Z*. *nevadensis*]	KDR16173
CRF receptor	73	0	0	4.79	6.91E-11	CRF receptor 2 [*S*. *mimosarum*]	KFM67631
FMRFamide receptor	515	11.16	11.13	1.21	2.00E-73	FMRFamide receptor [*T*. *castaneum*]	NP001280540
GnRHR-like-1	355	0	0	1.21	0.00E+00	GnRHR-like [*M*. *nipponense*]	AHB33640
GnRHR-like-2	349	0	0	0.56	0.00E+00	GnRHR-like [*M*. *nipponense*]	AHB33640
Leptin receptor	133	15.94	19.36	28.85	2.00E-77	Leptin receptor protein [*E*. *sinensis*]	ADV57398
NPF/PrRP receptor	96	0	4.6	0	5E-32/3E-46	NPF receptor [*A*. *gambiae*]/PrRP receptor [C. floridanus]	ABD96049/EFN70952
PDHR	82	0.46	0.28	6.73	8.49E-06	PDF receptor [*D*. *yakuba*]	XP002100255
SK receptor	98	2.79	1.38	0	6.00E-18	Perisulfakinin receptor [*T*. *castaneum*]	AAX56942
Tachykinin-like peptide receptor	189	4.85	16.88	0	3.00E-66	Tachykinin receptor like GPCR protein [*R*. *maderae*]	CAC36957

Abbreviations: ACP, AKH/corazonin-related peptide receptor; AST, Allatostatin; DH, Diuretic hormone; CRF, Corticotropin-releasing factor; GnRHR, Gonadotropin-releasing hormone receptor; NPF, neuropeptide F; PrRP, Prolactin-releasing peptide; PDH, Pigment-dispersing hormone; PDF, Pigment-dispersing factor; SK, Sulfakinin; GPCR, G protein-coupled receptor; Es, Eyestalk; CNS, Central nervous system; Ov, Ovary; FPKM, Fragments Per kb per Million fragments.

The total length of peptide, transcription sites with FPKM value, lowest E-value, BLASTx hits, and Genbank accession number of BLAST hit proteins are indicated.

#### Bursicons (Bur-α and -β), crustacean cardioactive peptide (CCAP), eclosion hormone (EH), pigment-dispersing hormone (PDH) and diuretic hormones: calcitonin-like diuretic hormone (CLDH) and diuretic hormone 45 (DH45)

**Fig 2:**
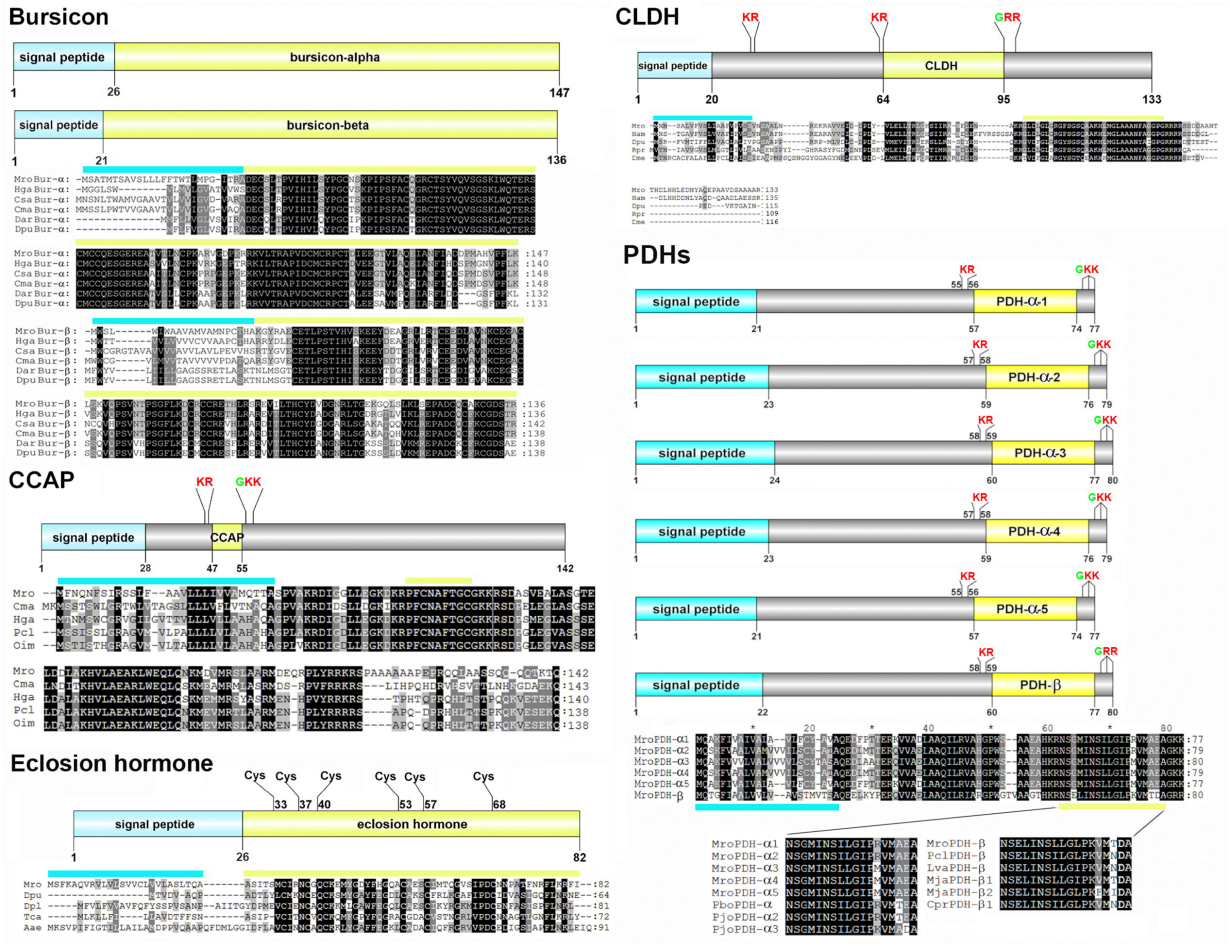
Molecular characterization of *Macrobrachium rosenbergii* bursicon, CCAP, CLDH, eclosion hormone, and PDHs. The schematic diagrams show the organization of putative neuropeptide precursors of *M*. *rosenbergii*, including signal peptides (blue), putative proteolytic cleavage sites (red), C-terminal glycine residues responsible for amidation (green), Cys residues and putative neuropeptides (yellow). Precursor sequence alignments are shown with site of putative neuropeptide denoted with a yellow bar. Conserved amino acids are shown in black shading while similar amino acids are shown with grey shading. Mro, is *M*. *rosenbergii* and for other species abbreviations see S1A Table.

Bursicon alpha and bursicon beta precursor transcripts were identified. The precursor proteins for Mro-Bur-α and Mro-Bur-β are organized with N-terminal signal peptides of 26 and 21 amino acids, respectively, followed by predicted bioactive peptides (Fig 2). Alignment of the Mro-Bur -α and -β with other crustacean species shows high spatial conservation of cysteines and strong similarity within each group (Fig 2). Mro-Bur-α and Mro-Bur-β precursor proteins showed between 73–78% and 54–63% similarity to other crustacean bursicons, respectively.

A single 142-amino acid, full-length CCAP precursor protein was deduced from a *M*. *rosenbergii* unigene (Fig 2). Mro-CCAP prepropeptide contains a 28-amino acid signal peptide followed by dibasic (K_45_R) and (K_57_K) cleavage sites that would release a bioactive CCAP peptide, 'PFCNAFTGC-NH2'. Comparison of the Mro-CCAP precursor with other arthropod CCAP sequences indicates high amino acid similarity, especially within the CCAP mature peptide sequences (100%).

A full-length EH precursor protein was deduced from a *M*. *rosenbergii* unigene (Fig 2). Mro-EH has a predicted 26-amino acid signal peptide and 6 spatially conserved cysteine residues with EH of other arthropod species, including within the active peptide region which has an overall 37–43% similarity.

Two types of PDH were identified in our study, PDH-α and -β. There were five isoforms of Mro-PDH-α precursors (possibly due to alternative splicing) comprising 77–80 amino acids (Fig 2). Based on the presence of enzymatic cleavage sites, both Mro-PDH-α-1 and -2 precursors would release an identical active peptide (NSGMINSILGIPRVMAEA-NH2) whereas Mro-PDH-α-3-5 would release a slightly different active peptide (NSGMINSLLGIPMVMAEA-NH2). For Mro-PDH-β, its precursor is composed of 80 amino acids with two dibasic cleavage sites (K_58_R and R_79_R), which would be cleaved to give rise to the bioactive peptide sequence (NSELINSLLGLPKVMTDA-NH2). An alignment of the active PDH-α and -β peptides shows high similarity of Mro-PDHs with other crustacean PDHs (Fig 2).

**Fig 3:**
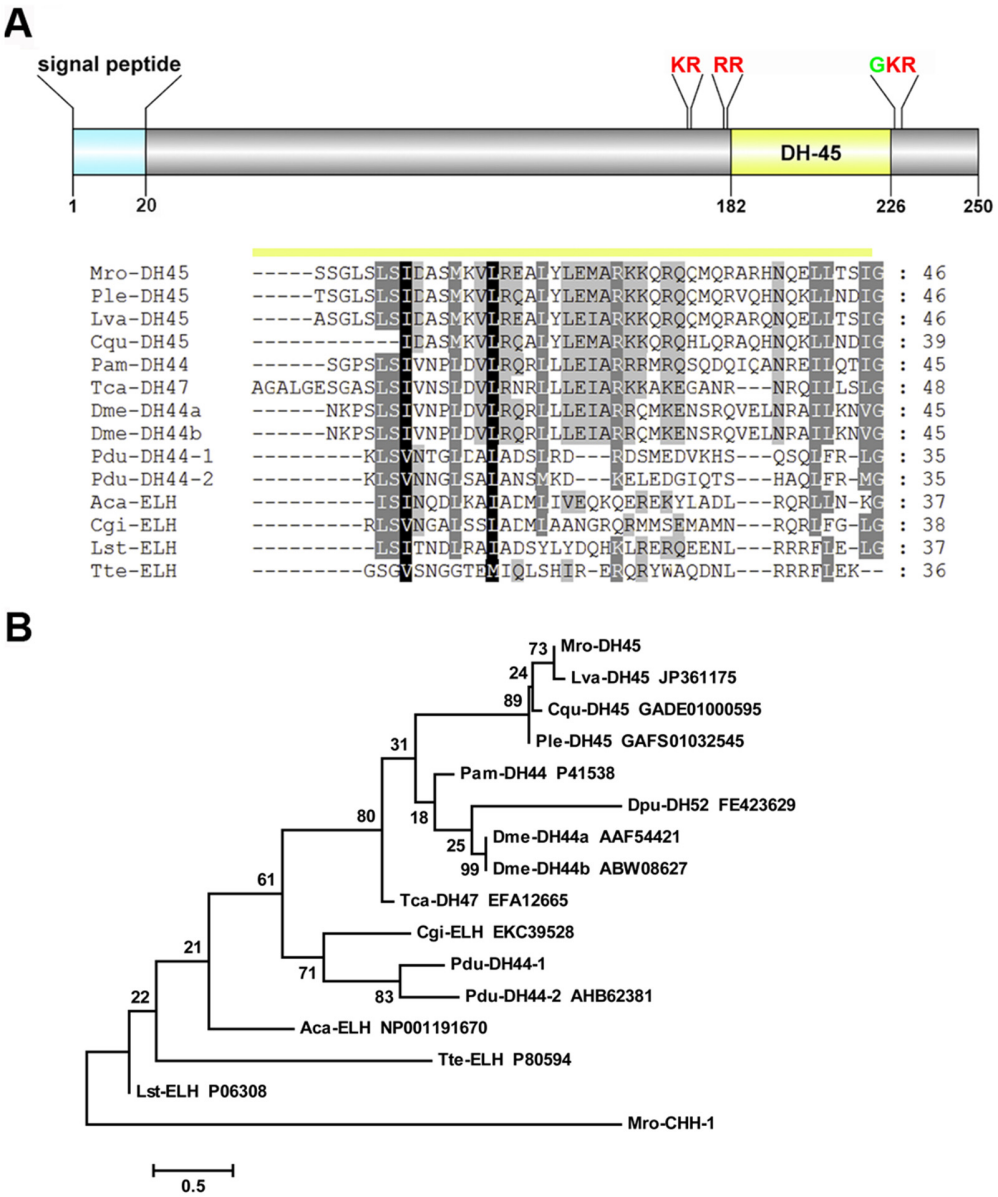
Molecular characterization of *Macrobrachium rosenbergii* DH45. (A) Schematic diagram showing the organization of DH45 neuropeptide precursor of *M*. *rosenbergii*, including signal peptide (blue), putative proteolytic cleavage sites (red), C-terminal glycine residues responsible for amidation (green), and putative neuropeptide (yellow). Alignment of DH45, DH44, and ELH active peptides is shown. Mro, is *M*. *rosenbergii* and for other species abbreviations see S1A Table. Conserved amino acids are shown in black shading while similar amino acids are shown with grey shading. (B) Phylogenetic tree of DH45, DH44, and ELH active peptides based on maximum likelihood estimation. Mro-CHH peptide sequence is used as a 'non-related' peptide for confirmation of phylogenetic tree reliability. Scale bar represents the number of amino acid substitutions per site.

A putative full-length CLDH precursor protein was deduced from a *M*. *rosenbergii* unigene (Fig 2). Mro-CLDH has 3 cleavage sites (K_30_R, K_63_R, and R_97_R) that could release a bioactive Mro-CLDH comprising 31 amino acid residues (GLDLGLGRGFSGSQAAKHLMGLAAANFAGGP-NH_2_). Alignment of the Mro-CLDH precursor protein with CLDH of other species revealed high amino acid conservation, particularly within the active region (Fig 2). The active region shares highest sequence similarity to lobster *Homarus americanus* CLDH (100% identity), and between 83–93% similarity to other arthropod CLDHs. Mro-CLDH is evolutionally close to the diuretic hormone 31 (DH31) in insects, and is known to be structurally related to calcitonin [48]. In addition, we could predict the Mro-DH45 precursor protein (Fig 3A). The precursor consists of a 20-residue N-terminal signal peptide and three proteolytic cleavage sites (K_170_R, R_180_R, and K_228_R). The predicted bioactive peptide is composed of 45 amino acids that is amidated (SSGLSLSIDASMKVLREALYLEMARKKQRQQMQRARHNQELLTSI-NH2). BLAST searches and multiple sequence alignment indicate that Mro-DH45 is homologous to other crustacean sequences within the NCBI shotgun transcriptome sequences, including *Pontastacus leptodactylus*, *Litopenaeus vannamei*, and *Cherax quadricarinatus* (Fig 3A). Their bioactive regions align strongly with other non-crustacean mature DHs, particularly the *T*. *castaneum* DH47 (50% identity to Mro-DH45) and *P*. *americana* DH44 (45% identity to Mro-DH45), and with some similarity to the molluscan egg-laying hormones (ELHs; 22% identity between *A*. *californica* ELH and Mro-DH45). Phylogenetic analysis shows that Mro-DH45 forms a branch with other decapod crustacean DH45 (89% bootstrap support; Fig 3B). A DH receptor transcript was identified within *M*. *rosenbergii* eyestalk transcriptome (Table 4).

#### Neuroparsin (NP), neuropeptide F (NPF), SIFamide and sulfakinin (SK)

**Fig 4:**
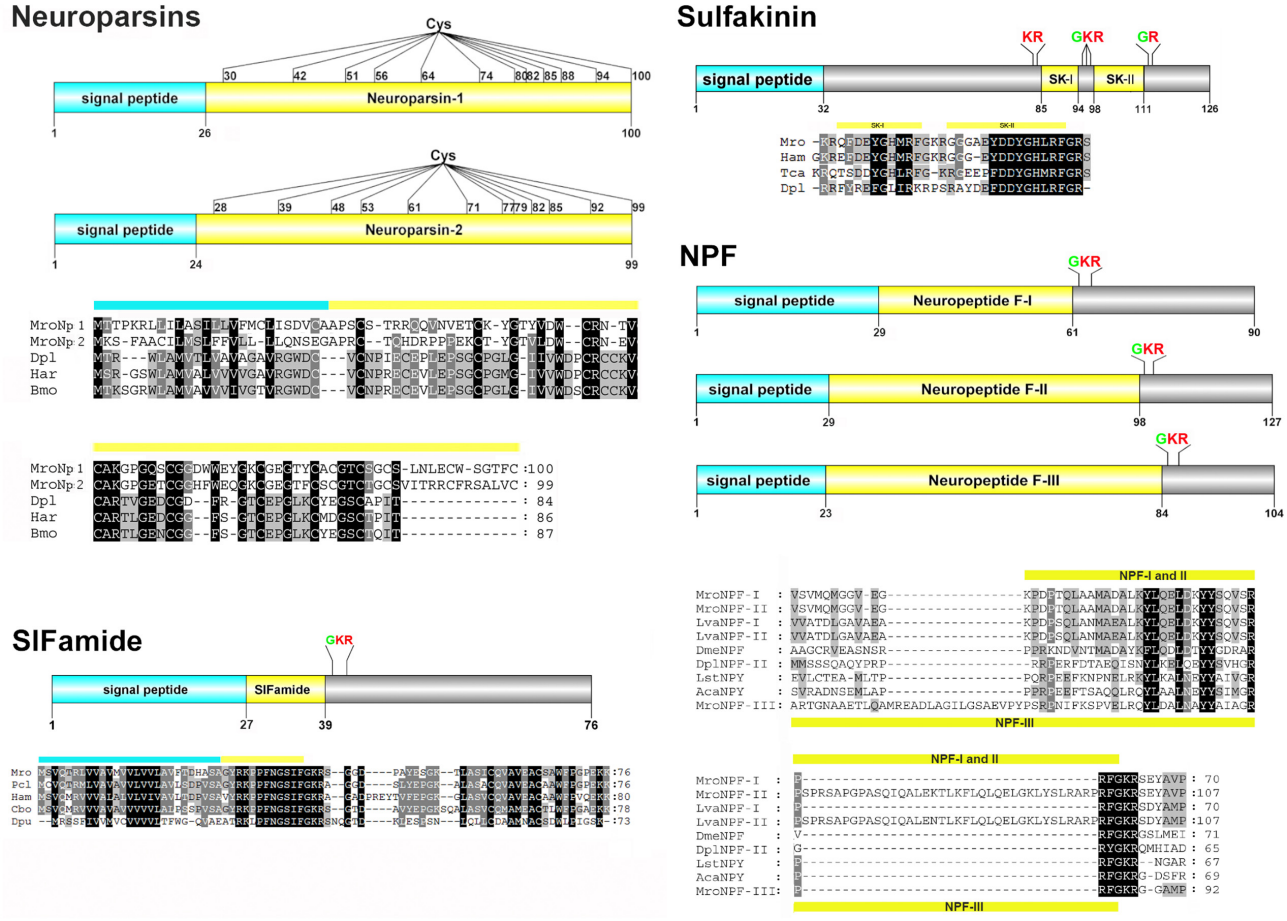
Molecular characterization of *Macrobrachium rosenbergii* neuroparsin, NPF, SIFamide, and sulfakinin. The schematic diagrams show the organization of putative neuropeptide precursors of the *M*. *rosenbergii*, including signal peptides (blue), putative proteolytic cleavage sites (red), C-terminal glycine residues responsible for amidation (green), Cys residues, and putative neuropeptides (yellow). Precursor sequence alignments are shown with site of putative neuropeptide denoted with a yellow bar. Conserved amino acids are shown in black shading while similar amino acids are shown with grey shading. Mro, is *M*. *rosenbergii* and for other species abbreviations see S1A Table.

Two NP precursors were identified in our study, Mro-NP-1 and -2 (Fig 4). Mro-NP-1 and -2 contain 26 and 24 residue N-terminal signal peptides, respectively, followed by bioactive peptides. Alignment of Mro-NPs with that of other species revealed high conservation of some amino acids within the mature NP region, especially cysteine residues (Fig 4). The Mro-NP-1 and -2 precursor proteins share 50% sequence similarity with each other and 23–26% to NPs found in insects.

Three isoforms of NPF could be deduced from *M*. *rosenbergii* unigenes (Fig 4). Mro-NPF-I to-III were classified as long NPFs. Based on predictions, Mro-NPF-I and-II consist of a 29-amino acid signal peptide followed by the active peptide, cleaved via a single dibasic cleavage site (K_63_R for Mro-NPF-I and K_100_R for Mro-NPF-II). Mro-NPF-III is composed of a 23-amino acid signal peptide and a 60-amino acid active peptide, cleaved at a single dibasic cleavage site (K_86_R) (Fig 4). Mro-NPF-I and II active peptides show high sequence identity (>90%) with *L*. *vannamei* NPF-I and-II, while the Mro-NPF-III active peptide shows high similarity (66%) with mollusc neuropeptide Y (NPY; the vertebrate homolog of NPF known to regulate feeding [49]). A short NPF (sNPF) transcript was also identified from the eyestalk and CNS transcriptomes; the Mro-sNPF precursor is predicted to be cleaved at multiple sites to give rise to four active peptides (Table 3 and S1 File). A partial-length NPF receptor-like sequence was deduced from the *M*. *rosenbergii* CNS transcriptome (Table 4).

A full-length SIFamide precursor protein was deduced from a *M*. *rosenbergii* unigene (Fig 4). Mro-SIFamide is composed of a 27-amino acid signal peptide followed by a predicted SIFamide active peptide and a dibasic cleavage site (K_41_R). It is highly conserved with other crustacean SIFamides, particularly within the bioactive SIFamide peptide (Fig 4). The bioactive Mro-SIFamide shares 100% sequence similarity to the SIFamides in crayfish (*P*. *clarkii*) and crab (*C*. *borealis*).

A single 126-amino acid full-length sulfakinin precursor protein was deduced from a *M*. *rosenbergii* unigene (Fig 4). Analysis of the deduced prepropeptide demonstrated a 32-amino acid signal peptide followed by multiple cleavage sites (K_83_R, K_96_R, and R_113_) to release the mature peptides, Mro-SK-I and-II (QFDEYGHMRF-NH2 and GGGAEYDDYGHLRF-NH2, respectively). The bioactive sulfakinins display highest sequence similarity to *H*. *americanus* (>90% identity). Based on post-translational modification predictions, the first glutamic acid of Mro-SK-I is cyclized while the tyrosine residues in both Mro-SKs are sulfated (Table 3). A sulfakinin receptor was predicted from *M*. *rosenbergii* eyestalk and CNS transcriptomes (Table 4).

#### Crustacean hyperglycemic hormone (CHH)

**Fig 5:**
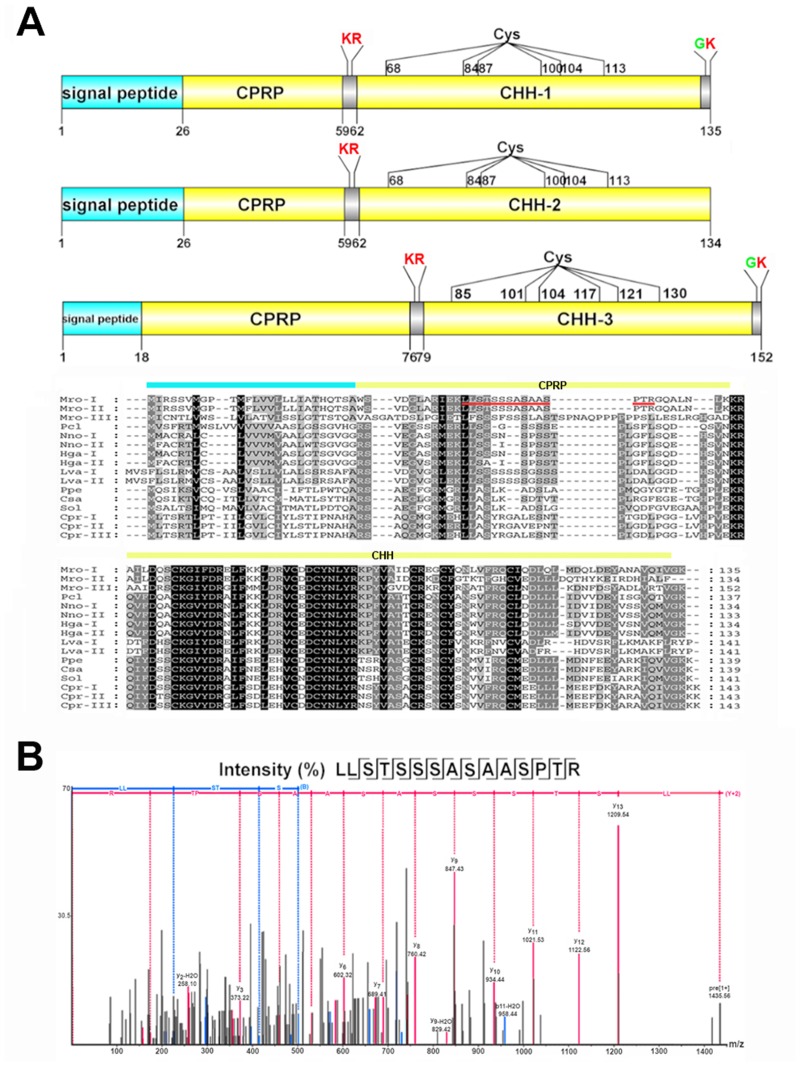
Molecular characterization of *Macrobrachium rosenbergii* CHH. (A) The schematic diagrams show the organization of putative CHH neuropeptide precursors of the *M*. *rosenbergii*, including signal peptides (blue), putative proteolytic cleavage sites (red), C-terminal glycine residues responsible for amidation (green), Cys residues, and putative neuropeptides (yellow). Precursor sequence alignments are shown with site of putative neuropeptide denoted with a yellow bar. Conserved amino acids are shown in black shading while similar amino acids are shown with grey shading. Mro, is *M*. *rosenbergii* and for other species abbreviations see S1A Table. (B) LC-MS/MS spectra showing fragment matching CHH precursor-related peptide (CPRP) which is indicated by red underline in (A).

Three isoforms of CHH were identified within the transcriptomes of female *M*. *rosenbergii*. Both Mro-CHH-1 and -2 have a 26-amino acid signal peptide, followed by a dibasic (K_60_R) cleavage site that would release the CHH precursor-related peptide (CPRP) and CHH mature peptides (72 and 73 amino acids in length, respectively) (Fig 5A). The Mro-CHH-3 comprises an 18-residue signal peptide and 2 cleavage sites at K_77_R and K_152_ (Fig 5A), releasing a 72-amino acid mature peptide. Mro-CHH-1 shares high sequence similarity with Mro-CHH-2 (81%), but only 47% similarity with Mro-CHH-3. All three Mro-CHHs show strong spatial conservation of cysteines with other crustacean CHHs (Fig 5A).

To examine the usefulness of the transcriptome-derived protein databases, we chose to further characterize the Mro-CHHs. Female *M*. *rosenbergii* eyestalk peptides were extracted for RP-HPLC using a 60 min gradient (S2 Fig): fractions were collected from 20 to 40 min, where the majority of abundant peaks were present. Fractions were subsequently combined for further analysis by LC-MS/MS. Among the identified proteins was a CHH precursor-related peptide, specifically matching to CPRP of Mro-CHH-1 and/or 2 precursor(s) (Fig 5B). Other abundant proteins identified with these fractions included hemocyanin and crustin (data not shown).

### Tissue-specific expression of neuropeptide genes

**Fig 6:**
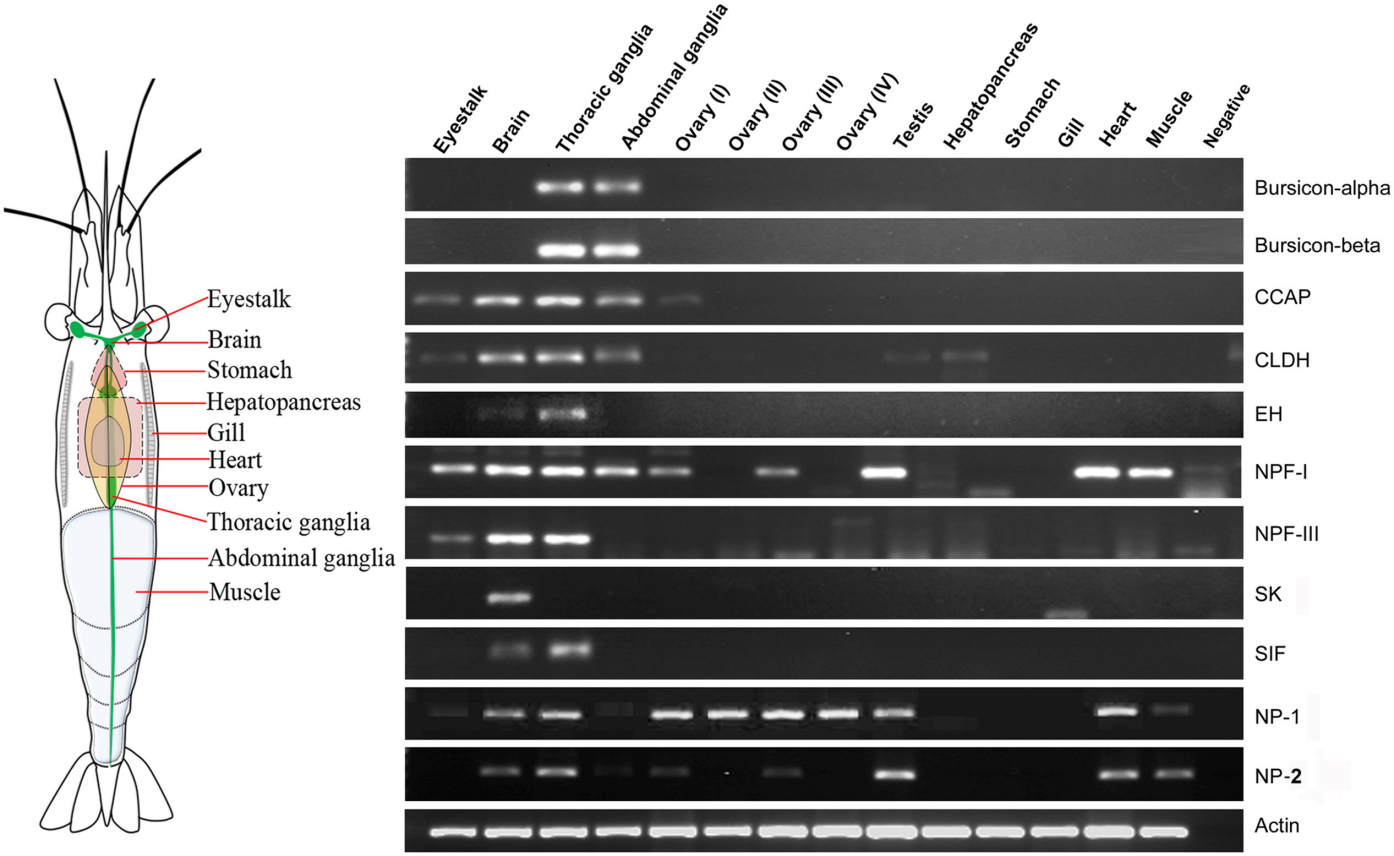
Tissue-specific expression of *Macrobrachium rosenbergii* neuropeptide genes, using RT-PCR. Expression of 11 neuropeptide genes using gene-specific primers, as well as the β-actin gene. PCR used cDNA derived from 13 tissues of female (including 4 stages of ovarian tissue) and 1 tissue of male (testis). Negative control represents no cDNA in PCR. CCAP, Crustacean cardioactive peptide; CLDH, Calcitonin-like diuretic hormone; EH, Eclosion hormone; NPF, Neuropeptide F; SK, Sulfakinin; SIF, SIFamide; NP, Neuroparsin.

After obtaining nucleotide sequences encoding *M*. *rosenbergii* neuropeptides, we investigated their expression in different tissues by RT-PCR in order to: (i) confirm that neuropeptide transcripts were synthesized in the eyestalk, CNS, and/or ovary; and (ii) to check whether neuropeptide genes were expressed in other tissues (Fig 6). RT-PCR showed that *Mro-Bur-α* and *-β* were expressed only in thoracic and abdominal ganglia. *Mro-EH* and *Mro-SIFamide* transcripts were detected in the brain and thoracic ganglia, although the level of *Mro-EH* in the brain was relatively low as indicated by a weak intensity amplicon. The *Mro-CCAP* gene was expressed in all regions of the CNS while relatively less expression was observed in the eyestalk and ovary stage I (not detected by the transcriptome sequencing). *Mro-CLDH* was expressed prominently in the CNS, as well as the eyestalk, testis, and hepatopancreas. *Mro-NPF-I* showed a strong level of expression in many tissues, except ovary stage II, IV, hepatopancreas, stomach, and gill. *Mro-NPF-III* appeared to have the highest level of expression in the brain and thoracic ganglion, but lower expression in the eyestalk. *Mro-sulfakinin* appeared to be specific to brain tissue. *Mro-NP-1* and *-2* had a similar pattern of tissue distribution but relative abundance in each tissue varied. *Mro-NP-1* was expressed in most tissues, except the hepatopancreas, stomach and gill. Likewise, *Mro-NP-2* was expressed within most tissues, except the eyestalk, hepatopancreas, stomach and gill.

## Discussion

In this study, we could identify neuropeptide transcripts by *in silico* searches of female *M*. *rosenbergii* eyestalk, CNS, and ovary transcriptomes, tissues that are known to directly influence growth and reproduction. In total, we have identified 37 transcripts encoding preproneuropeptides and 13 transcripts encoding neuropeptide receptors, some of which show CNS or eyestalk specificity.

### Transcriptomes of female *M*. *rosenbergii* eyestalk, CNS, and ovary

From the three tissues combined, a total of 78,110 unigenes were assembled and investigated. Only 33.4% of all unigenes assembled from combined transcriptomes matched with other known genes in databases, most of which represented crustaceans and insects. The reason for the low number of matched genes is that crustacean transcripts are relatively poorly represented in databases. Secondly, *de novo* assembly can sometimes produce artifact sequences that ultimately influence downstream analyses, for instance, reads mapping, sequence clustering, base-call error, and number of BLAST hits [35, 50]. Importantly, some of them might be novel genes that have never been identified, or represent genes containing non-conserved domains, which therefore could not be classified into known protein families. Not surprising, the majority of *M*. *rosenbergii* unigenes matched with *D*. *pulex*, for which a genome is available (the first for crustaceans) [51]. A comparison of the three individual *M*. *rosenbergii* transcriptomes (eyestalk, CNS, and ovary) indicated that the majority of unigenes appeared to be tissue-specific. Although eyestalk, CNS, and ovary show a distinct unigene content, there is no substantial difference at the level of protein functional domain annotation. Combining the representative unigene and Pfam comparison results, reveals that the three tissues have a significant number of tissue-specific transcripts belonging to similar protein families and therefore may conduct similar biological processes in different cell types (Fig 1D). Based on COG classification and GO assignment, most of unigenes were functionally categorized into the cellular and metabolic processes, a similar outcome to that of other crustacean transcriptome studies [19, 20, 23]. This is a good indication that genes associated with these processes are typically highly conserved throughout evolution and essential for multicellular organism survival.

### Prediction of neuropeptides from *M*. *rosenbergii* transcriptomes

Neuropeptides show evolutionary conservation since they are known to be critical for growth and reproduction, and as such, we used a targeted approach to search for *M*. *rosenbergii* neuropeptides. Eyestalk and CNS were used in this study because they are known to be primary sites from which the majority of crustacean neuropeptides are synthesized and released into the hemolymph [1, 32, 52]. From this study, targeted tBLASTn of a combined three-transcriptome dataset of *M*. *rosenbergii* revealed at least 21 transcripts (some indicated multiple isoforms) that matched with previously known crustacean preproneuropeptides [7, 9, 53]. Our predictions for precursor cleavage sites indicate that a total of 102 mature peptides may be released, along with a number of precursor-related peptides (Table 3). Similar *in silico* analysis has been performed on the copepod (*Calanus finmarchicus*) from embryo, early nauplii, late nauplii, early copepodite, pre-adult, and female adult transcriptomes, revealing 34 preprohormone and 18 hormone receptor transcripts [9]. In contrast to our study, they utilized whole animal RNA for library assemblies. Neuropeptide transcripts have also been discovered from whole animal RNA obtained from the ice krill, *Euphausia crystallorophias* [53], but the RNA was supplemented with the eyestalk RNA to supposedly increase the possibility of identifying neuropeptide transcripts. Our study identified a relatively higher number of neuropeptide isoforms, possibly caused by the different range of tissues and metabolic stages from which their libraries were constructed.

Since crustacean neuropeptides have also been identified from ovarian tissue based on immunolocalization and mass spectrometry [12, 54–57], we performed an additional *in silico* transcriptome investigation of the *M*. *rosenbergii* ovary. We could only find 2 preproneuropeptide transcripts in the ovary, including neuroparsin-1 and -2, which were also found in the eyestalk and CNS. The gonadotropin-releasing hormone (GnRH) is known as the master hormone controlling reproduction in vertebrates and some invertebrates [58, 59]. Several immunolocalization studies have revealed the existence of a GnRH-like peptide in the CNS and ovary of crustaceans [54–56]. In addition, a GnRH peptide was identified in the ovary of crayfish, *P*. *clarkii*, by RP-HPLC purification and N-terminal sequencing [57]. Despite of the identification of a GnRH receptor (Table 4), we could not yet find any GnRH-like transcript in the ovary transcriptome of *M*. *rosenbergii*, nor the other tissue transcriptomes. However, we could identify other members of the GnRH superfamily [60], including corazonin and red pigment-concentrating hormone (RPCH) transcripts, present in the eyestalk and CNS transcriptomes. Possibly, a transcript that encodes GnRH may be present yet not picked up in this study due to either low conservation, low expression level, or its half-life may be very short.

Bursicon, a highly conserved arthropod neuropeptide, plays an important role in the regulation of cuticle sclerotization (hardening and tanning) after ecdysis [61]. A functional unit of bursicon consists of two cystine knot subunits, bursicon-α and -β forming a heterodimeric protein that regulates the cells via a G protein—coupled receptor [62, 63]. We found in *M*. *rosenbergii* two CNS transcripts that encode bursicon-α and -β subunits, displaying high conservation with other crustacean bursicons. RT-PCR showed that *Mro-Bur-α* and *-β* transcripts are expressed in the thoracic and abdominal ganglia (Fig 6), a similar finding to that in *C*. *maenas*, where bursicons are only found in the thoracic ganglion but not in the eyestalk and brain [64]. Bursicons have been linked to the processes of molting and development since their expression levels vary throughout these physiological processes [64, 65].

The crustacean cardioactive peptide (CCAP) is a highly conserved amidated nonapeptide with the primary sequence 'PFCNAFTGC-NH2' [32, 66]. The Mro-CCAP mature peptide shares this sequence, while the Mro-CCAP precursor-related peptides are not identical yet still most similar to that of other decapods (Fig 2). Besides being a potent cardioexcitator peptide [67–69], CCAP is known to exhibit pleiotropic functions in other species, including modulation of the pyloric rhythm in crabs [70], regulating oviduct contraction in locusts [71], inducing pigment dispersion in crabs [72], modulating the buccal feeding network in pond snails [73], and has been implicated in ecdysis [74, 75]. CCAP gene expression and its peptide have been found throughout the decapod CNS [75–77]. In this study, the *Mro-CCAP* transcript was found in both the eyestalk and CNS transcriptomes (Table 2), supported by RT-PCR which indicated widespread expression throughout the eyestalk, brain, thoracic ganglia, and abdominal ganglia (Fig 6). Expression was also observed, but with lower intensity, in the ovarian tissue, suggesting a role for CCAP in reproduction in *M*. *rosenbergii*.

Another neuropeptide associated with ecdysis in crustaceans is the eclosion hormone (EH). The function of EH has been intensively studied in insects (for review, see [78, 79]) but not in crustaceans, although a number of EHs have been reported in crustacean species [25, 80]. In our study, the *Mro-EH* transcript was discovered in both the eyestalk and CNS transcriptomes. Based on multiple sequence alignment, the Mro-EH mature peptide displays six conserved cysteine residues that may be responsible for the formation of three-disulfide bridges [81, 82]. RT-PCR showed that the *Mro-EH* gene was expressed in the brain and thoracic ganglia, but not in the other tissues, including the eyestalk, probably due to low or no expression.

Diuretic hormones (DHs) regulate water balance in order to maintain homeostasis in insects [83]. In our study, we found two transcripts encoding for DHs, calcitonin-like and corticotropin-releasing factor (CRF)-like DHs. The calcitonin-like DH (CLDH) mature peptide is highly conserved throughout crustacean and insect CLDHs. Besides observed expression of *Mro-CLDH* in the nervous tissues, it could be detected in the testis and hepatopancreas, suggesting a role for the encoded peptide in the physiological activities of these organs. We also present a crustacean DH-45 (DH45) precursor, which helped to reveal other highly conserved crustacean DH45, in *L*. *vannamei*, *P*. *leptodactylus*, and *C*. *quadricarinatus*. The DH45 peptide shows similarity with insect DH44s which are known to be members of the corticotropin-releasing factor (CRF)-like peptide family, which influence water balance and fluid secretion [84]. DH44 is thought to be the ortholog of molluscan ELH [85, 86], a hormone that induces egg-laying in the gastropod mollusc *Aplysia* [87, 88] and possibly other molluscs. Hence, we compared various crustacean DH45 with various insect DH44 and molluscan ELH, showing the conservation specifically within several amino acid residues (Fig 3A). Phylogenetic tree analysis supports a close evolutionary relationship of CRF-like DHs among insects and crustaceans, and with ELH considering that ELH-like peptides are diverse (besides approximate length and several residues). Previous immunolocalization studies have demonstrated the presence of an ELH-like peptide in crustaceans, including the black tiger shrimp *P*. *monodon* and *M*. *rosenbergii* [89, 90]. Meanwhile, injection of molluscan ELH could decrease the time of ovarian maturation, also in *M*. *rosenbergii* [90]. Whether the crustacean DH45 is the ELH-like peptide or even has a role in reproduction, requires further investigation.

The pigment-dispersing hormone (PDH) is a neurohormone that is released primarily from the eyestalk and influences pigment dispersion in the body integument and compound eyes of crustaceans [91]. In this study, we could identify five isoforms of Mro-PDH-α (Mro-PDH-α-1 to -5) and one isoform of Mro-PDH-β (Fig 2). All *Mro-PDH-α* transcripts appear to be exclusive to the eyestalk, while the *Mro-PDH-β* is present in both the eyestalk and CNS. Within these isoforms, there was no transcript corresponding to the previously identified *M*. *rosenbergii* PDH-α that encodes an active peptide of 'NSGMINSILGIPKVMAEA-NH2 [92]. Based on our alignment of the newly identified isoform sequences, including 5' and 3' untranslated regions (UTR) (data not shown), the nucleotides encoding the C-terminal region of precursors as well as 3'UTRs of *Mro-PDH-α-1* and *-2* were almost identical (only a single nucleotide difference) and we could also observe this similarity pattern between *Mro-PDH-α-4* and *-5*. This result suggested that the isoforms of *MroPDH-α* may be derived from alternative splicing. Also, this may reflect the presence of a single nucleotide polymorphism in the population, since tissue transcriptome RNAs were combined from multiple individuals. In contrast, *D*. *pulex* has only a single *pdh*-gene which encodes a beta-type PDH whilst no alpha-type PDH has been found [80]. The precise number of Mro-PDH variants within *M*. *rosenbergii* may be fully explored through genome sequencing and expansion of tissue transcriptomes available. Despite the presence of multiple *Mro-PDH*, we identified only one *Mro-PDH* receptor (PDHR), present within the eyestalk, CNS, and ovary, implicating it directly in some kind of neuromodulator role, supported by another study in the crab, *Cancer productus* [93]. Also, the PDH homolog in *C*. *elegans*, PDF [pigment dispersing factor) is known to regulate egg-laying [94]. A role for PDH in controlling egg laying in crustaceans has not yet been reported and this should be explored further.

Neuroparsins (NPs) were initially identified as anti-gonadotropic agents in insects [95]. Subsequent investigations showed that NPs have more diverse roles, including anti-juvenile, antidiuretic, hypertrehalosemic and hyperlipemic effects (for review, see [96]). Typically, a single NP precursor could be identified in a single species of most insects, except in the locust in which NP variants were identified [96]. In *D*. *pulex*, an NP gene was identified that encodes a 78-amino acid NP peptide containing 14 cysteine residues possibly forming 7 disulfide bridges [80]. NPs in other crustacean species were also deduced from expressed sequence tags (EST) searches and three NP isoforms has been predicted in *C*. *rogercresseyi* [25]. In our study, two transcripts encoding for NP prepropeptides, *Mro-NP-1* and *-2*, were found. Both encode mature NPs consisting of 12 cysteine residues (possibly forming 6 disulfide bridges) which is typical of other decapod NPs [7, 25, 38]. However, Mro-NP-1 and -2 peptide precursors share only 50% amino acid similarity and their 5'- and 3'-UTRs are not similar (data not shown). Therefore, it seems likely that *Mro-NP* transcripts are derived from separate genes. In the ovary, the relative levels of *Mro-NP-1* and *-2* gene expression appear to vary; *Mro-NP-1* expression was consistent throughout ovarian development (stages I to IV), while *Mro-NP-2* expression was only detected within ovarian development stages I and III. Despite this, their presence suggests a role in ovarian development for this species. Its additional presence in multiple other tissues is consistent with that found in the spiny lobster (*Jasus lalandii*), showing NP distribution within the eyestalk X-organ, brain, sub-esophageal ganglia, thoracic ganglia, ovary, and heart [97].

Neuropeptide F (NPF) is specific to invertebrates and ancestrally related to vertebrate neuropeptide Y (NPY) that is a potent stimulator controlling feeding behavior, energy homeostasis, and reproduction. Unlike NPY, NPF features a C-terminal RxRF-NH2 [98, 99]. Three *Mro-NPF* transcripts were identified in our study. Previously, two isoforms of NPFs, NPF-I and II, could be identified in crustacean species, including *C*. *finmarchicus* [9], *E*. *crystallorophias* [53], *L*. *vannamei* and *M*. *marginatus* [100]. The only difference between their NPF-I and-II is that NPF-II contains an insertion, which is also observed between Mro-NPF-I and-II. Since the NPF-I and-II in *D*. *pulex* are produced from alternative splicing of an *npf*-gene [80], this appears to be conserved with the decapods, although confirmation is required through genome analysis. The presence of these two NPFs in many species suggests significant roles for both isoforms in crustaceans. The Mro-NPF-III exhibits a vastly different amino acid sequence to MroNPF-I and-II (32% and 26% identity, respectively). Although still NPF-like, it also has similarity to molluscan NPYs based on sequence alignment and subsequent phylogenetic analysis (data not shown). Interestingly, while *Mro-NPF-I* was expressed in various tissues, *Mro-NPF-III* expression appears restricted to neuronal tissues (eyestalk, brain, and thoracic ganglia). This contrasts gene expression results in penaeid shrimps, where NPF-I and-II are both detected in neuronal tissues, while only NPF-I is additionally found in the midgut, suggesting its dual role as a paracrine/autocrine and endocrine hormone [100]. Indeed, non-nervous tissue, including the heart and muscle, can be located very closely to ganglia/neurosecretory cells [101, 102]. Nevertheless, confirmation through investigation of protein expression by mass spectrometry or immunolocalization using isoform-specific anitibodies is required. A functional test of crustacean NPFs has been carried out in *L*. *vannamei*, revealing an orexigenic activity of Lva-NPF-I [100]. Since there is high similarity between *M*. *rosenbergii* and *L*. *vannamei* NPF-I, it is likely that Mro-NPF-I may also have appetite stimulant effects in *M*. *rosenbergii*. In insects, NPF plays roles in feeding, learning, responses to stress [103], ecdysteroidogenesis [104], and reproduction [103]. In addition, the single Mro-short NPF (Mro-sNPF) precursor is predicted to release four active peptides, including ones that contain the sNPF consensus C-terminal sequence 'xPxLRLRF-NH2' [99]. Two sNPF precursors have been identified in *E*. *crystallorophias*, which are cleaved and give rise to several active sNPF peptides [53]. Insect sNPFs are thought to be involved in the regulation of feeding, circadian rhythm as well as sleep [99, 105–107] while the function of crustacean sNPF is still not known.

SIFamide is a conserved short neuropeptide that has been identified in the CNS of many insects and crustaceans [108]. We identified a single transcript encoding the Mro-SIFamide prepropeptide in both the eyestalk and CNS transcriptomes. The mature Mro-SIFamide peptide is a [Gly^1^]-SIFamide, a feature that seems to be exclusive in decapods [32, 108], while its function in these species is poorly understood. The spatial distribution of SIFamide in the nervous tissue of marbled crayfish indicated its role in visual processing [109]. In prawns, SIFamide modulates aggression in male *M*. *rosenbergii* as indicated by behavioral changes upon [Gly^1^]-SIFamide injection [110]. As determined in our study, *Mro-SIFamide* is expressed within the nervous tissue, including brain and thoracic ganglia (Fig 6). However, we could not detect the expression of *Mro-SIFamide* in the eyestalk and abdominal ganglia by RT-PCR, possibly due to its low expression in these tissues.

Sulfakinin (SK) is a neuropeptide found in arthropods that is structurally and physiologically homologous to mammalian gastrin/cholecystokinin peptides (for review, see [111]). The deduced Mro-SK precursor should produce two mature SK peptides, Mro-SK-I and-II. Mro-SK-I exhibits a classical SK characteristics, including the consensus sequence 'XXYGHMRF-NH2' (where X are acidic residues either E or D), an N-terminal pyroglutamic acid, a sulfated tyrosine residue, and C-terminal amidation. Mro-SK-II shares a similar organization to decapod glycine-rich SKs [112–114], including a glycine-rich N-terminus, a substitution for C-terminal methionine (by L/I) and disulfation [113]. In insects, the function of SK is more well established, and includes the regulation of food intake [115, 116], the release of digestive enzymes [117], and gut contraction [118]. In crustaceans where less is known, SKs appear to play a role as a neuromodulator [112] and cardioactive agent [114]. SKs have been reported in the CNS of many crustaceans [80, 112–114]. While the SK peptide was detected throughout the CNS of tiger prawn (*P*. *monodon*) [112], our study by RT-PCR could detect the *Mro-SK* gene expression only within the brain tissue. The difference may relate to spatial differences between peptide and transcript.

Crustacean hyperglycemic hormone (CHH) is produced from the XO-SG complex in the eyestalk and primarily controls carbohydrate metabolism in crustaceans [119]. However, the presence of CHH in extra-eyestalk tissues (including CNS, peripheral neurons, pericardial organ, heart, antennal gland, and gill) and its other roles, such as modulating reproduction, have been reported [119, 120]. In our study, we identified three isoforms of Mro-CHH (Fig 5). Two alternatively spliced isoforms had previously been identified in *M*. *rosenbergii*; CHH (short form) and CHH-L (long form) [120], called CHH-1 and -2, respectively, in our study. We found that both isoforms are present within eyestalk and CNS transcriptomes. Despite inducing hyperglycemia, CHH is pleiotropic and can also exhibit an osmoregulartory role [121] as well as an inhibitory role for molting and methyl farnesoate synthesis [122]. CHH genes and associated peptides appear to be polymorphic, where at least two isoforms could be present in a single species [123]. Up to now, more than 24 CHH-like genes have been found in several crustacean species (for reviews, see [119, 124]), including three within *D*. *pulex* [80]. Therefore, it was not surprising that we found a third CHH isoform in *M*. *rosenbergii*, *Mro-CHH-3*, that is present in the eyestalk but not the CNS. It would be of interested to investigate its function in relation to the other isoforms.

The usefulness of the transcriptome-derived protein databases was tested through LC-MS/MS of eyestalk peptides and identification of Mro-CHH-1 and/or 2 precursor peptides, but not Mro-CHH-3, possibly due to low level peptide expression within that tissue. Also, as our protocol did combine RP-HPLC fractions prior to MS/MS analysis, spectra obtained may not have been of appropriate intensity for its identification. Despite this, we believe that our transcriptome-derived protein libraries will be of value for future peptidomic studies.

## Conclusions

The results obtained from this study provide a greater understanding of the neuropeptidergic signaling system of *M*. *rosenbergii*, an important cultured crustacean species. The majority of neuropeptide precursors assembled and described here have not been described previously for this species. Inevitably, understanding of neuropeptides that regulate growth and reproduction at a molecular level is very important, and may provide us with the tools for animal manipulation that could reliably increase the numbers of cultured animals.

## Supporting Information

S1 Fig Clusters of orthologous groups (COGs) classification and gene ontology (GO) assignment. (A) Distribution of COG classification for the transcriptomic unigene sequences of *M*. *rosenbergii*. (B) Histogram presentation of GO terms of all unigene sequences of *M*. *rosenbergii*, which are classified into 3 main categories: biological process, cellular component and molecular function.
(TIF)

S2 Fig Representative RP-HPLC chromatogram of eyestalk peptide extract at absorbance 210 nm. Fractions within elution time 20–40 min were collected and pooled (red double-headed arrow) for LC-MS/MS analysis.
(TIF)

S1 Table Species abbreviations and Genbank accession numbers. (A) Abbreviations and species names used for multiple sequence alignments and phylogenetic tree. (B) Available Genbank accession numbers of peptides used in neuropeptide alignments are provided.
(XLSX)

S2 Table Summary of transcriptome sequencing results of *Macrobrachium rosenbergii* eyestalk, CNS, ovary, and combined datasets. (XLSX)

S1 File Amino acid sequences for all *Macrobrachium rosenbergii* preproneuropeptides and receptors. (FASTA)

S2 File Nucleotide sequences for all *Macrobrachium rosenbergii* preproneuropeptides and receptors. (FASTA)
