# Supplementary figures and images for: In silico Neuropeptidome of Female Macrobrachium rosenbergii Based on Transcriptome and Peptide Mining of Eyestalk, Central Nervous System and Ovary
Source: PLoS One. 2015 May 29;10(5):e0123848. doi: 10.1371/journal.pone.0123848 (PMC4449106; doi:10.1371/journal.pone.0123848)

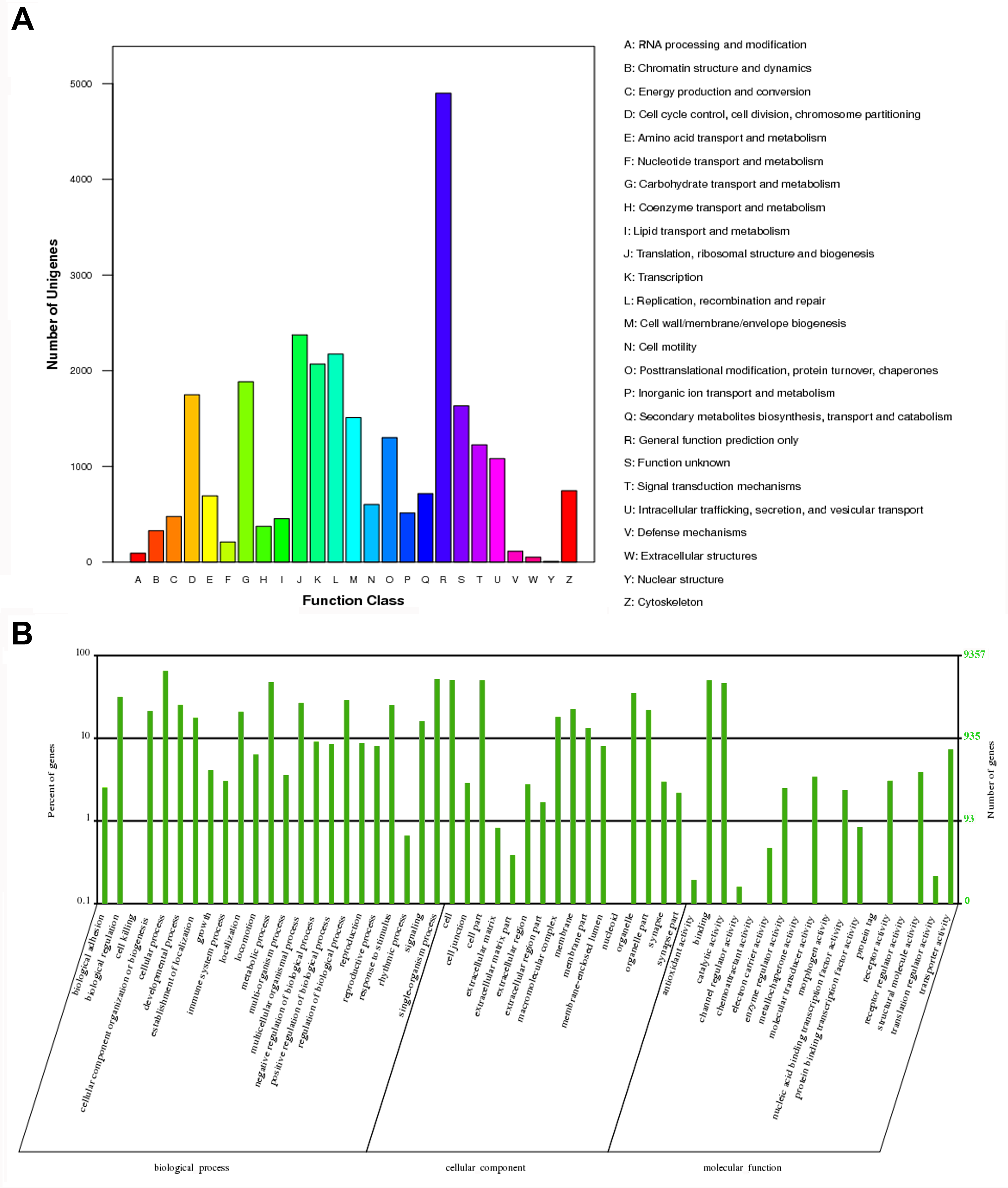

Supplement: S1 Fig — (A) Distribution of COG classification for the transcriptomic unigene sequences of M. rosenbergii. (B) Histogram presentation of GO terms of all unigene sequences of M. rosenbergii, which are classified into 3 main categories: biological process, cellular component and molecular function. (TIF) [file pone.0123848.s001.tif]

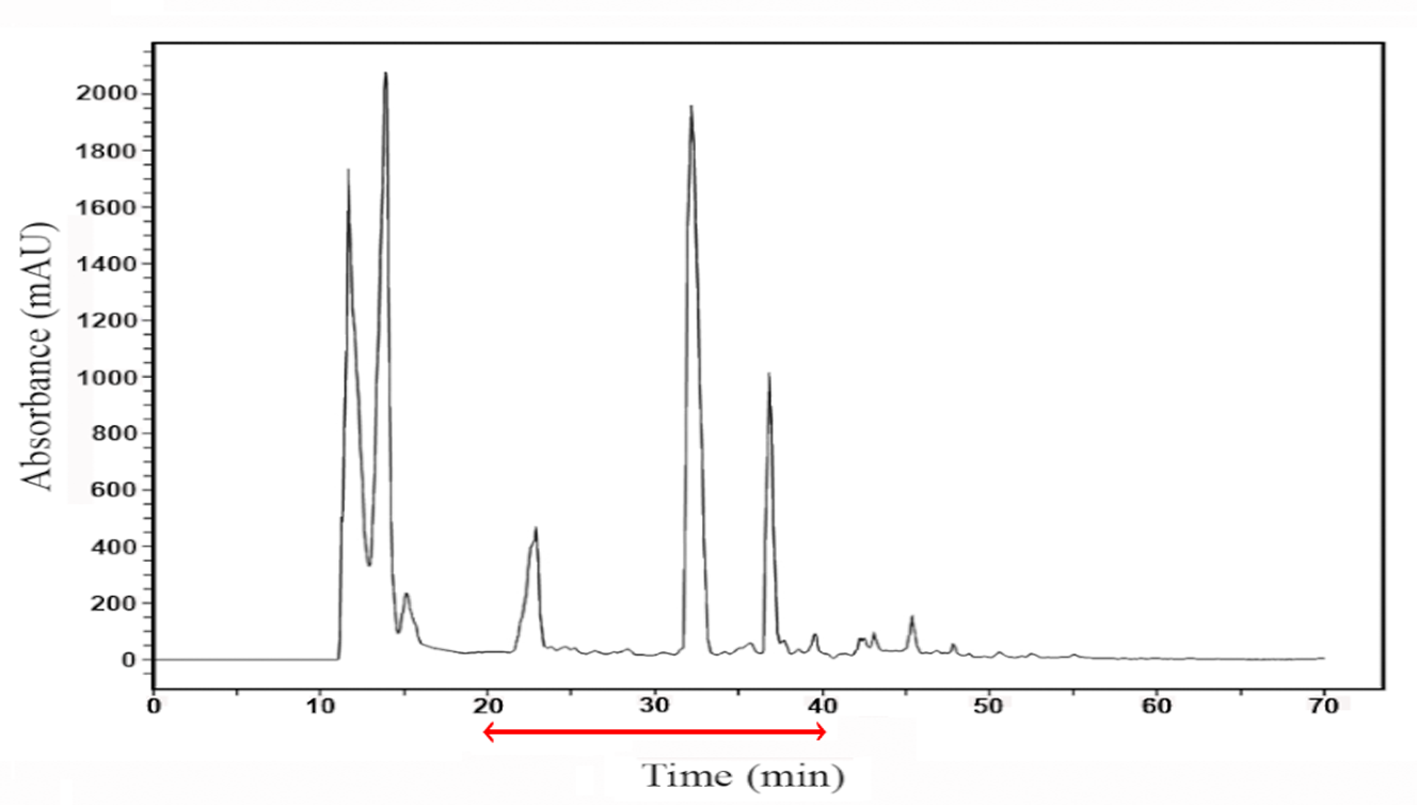

Supplement: S2 Fig — Fractions within elution time 20–40 min were collected and pooled (red double-headed arrow) for LC-MS/MS analysis. (TIF) [file pone.0123848.s002.tif]
